# Therapeutic effect of *Tripterygium hypoglaucum (H. Lév.) Hutch.* extract on psoriasis-like skin inflammation correlated with gut microbiota homeostasis in mice

**DOI:** 10.3389/fphar.2026.1822819

**Published:** 2026-05-12

**Authors:** Zhimin Wang, Huan Cui, Hong Huang, Peilian Zhang, Lihua Yin, Guangyun Luo, Qin Li, Yiwen Zhang, Lingzhi Meng, Wenyu Chang, Xiang Li, Xuesong Yang, Jianzhou Ye

**Affiliations:** 1 First School of Clinic Medicine, Yunnan University of Traditional Chinese Medicine, Kunming, Yunnan, China; 2 Department of Dermatology, First Affiliated Hospital of Yunnan Traditional Chinese Medicine University, Kunming, Yunnan, China; 3 Department of Dermatology and Venereology, Linquan County People’s Hospital, Fuyang, Anhui, China

**Keywords:** 16S rrna, gut microbiota, intestinal contents metabolomics, psoriasis, *Tripterygium hypoglaucum* hutch

## Abstract

**Background:**

*Tripterygium hypoglaucum (H. Lév.) Hutch*. (THH) exerts anti-inflammatory and immunosuppressive effects against psoriasis. However, the extent to which and the mechanisms by which it ameliorates psoriasis-like dermatitis through modulation of the gut microbiota-metabolite axis remain unclear.

**Methods:**

This study investigated the therapeutic effects and underlying mechanisms of THH in a murine model by employing 16S rRNA gene sequencing, intestinal metabolomic profiling, fecal microbiota transplantation (FMT), and co-housing experiments.

**Results:**

Our results demonstrated that THH treatment significantly decreased PASI scores, alleviated epidermal hyperplasia and skin inflammation, and reversed IMQ-induced gut dysbiosis by restoring the Firmicutes/Bacteroidota ratio and modulating the abundance of beneficial and pathogenic bacteria. Metabolomic analysis revealed that THH normalized multiple metabolic pathways disturbed by IMQ, including arachidonic acid metabolism, sphingolipid metabolism, and primary bile acid biosynthesis. Correlation analyses further revealed significant associations among the altered gut microbiota, key metabolic pathways, and psoriasis-related phenotypic indices. Moreover, FMT from THH-treated mice conferred significant anti-psoriatic efficacy. Similarly, co-housing experiments resulted in the alleviation of skin lesions, reduction of spleen weight, and downregulation of inflammatory cytokines.

**Conclusion:**

These findings demonstrate that THH attenuates psoriasis-like dermatitis partly by reshaping gut microbiota composition and regulating key metabolic pathways, supporting a potential gut-targeted therapeutic strategy for psoriasis.

## 1 Introduction

Psoriasis represents a chronic, immune-mediated dermatological disorder affecting approximately 125 million individuals worldwide. Clinically, it manifests as erythematous, scaly plaques and systemic inflammation (Lebwohl, 2018; Greb et al., 2016), characterized histologically by hyperkeratosis, erythema, and scaling, alongside immunological features such as neutrophil microabscesses and the infiltration of γδ+ T cells and Th17 cells into the skin. The pathophysiology is marked by epidermal hyperplasia, abnormal differentiation, vasodilation, and immune cell infiltration (Takeshita et al., 2017; Korman, 2020). Excessive activation of the IL-23/IL-17 axis constitutes a central pathogenic mechanism. Environmental and genetic factors activate plasmacytoid dendritic cells, which, along with dendritic cells and macrophages, produce IL-23. This cytokine promotes Th17 cell differentiation and subsequent IL-17 secretion. IL-17 synergizes with TNF to induce keratinocyte proliferation and chemokine production, thereby recruiting additional Th17 cells to inflammatory sites. This self-perpetuating feedback loop sustains chronic inflammation and epidermal hyperplasia (Puig et al., 2022). Although biologic agents targeting specific cytokine pathways have revolutionized treatment for severe cases, therapeutic limitations persist. Disease relapse frequently follows drug discontinuation, and an increasing number of patients exhibit refractoriness to all available therapies, substantially amplifying the psychosocial and economic burdens imposed by psoriasis on individuals and society.

Intestinal microbial flora is recognized as an indispensable “metabolic organ” that plays crucial roles in maintaining human health and initiating diseases, including digestion, nutrient absorption, energy supply, immune regulation, and disease resistance (Lin et al., 2018; Kelly et al., 2005; Xu et al., 2003). The intestinal microbial flora comprises trillions of microorganisms that colonize the gastrointestinal tract and are involved in many local and systemic processes (Wang et al., 2020; Mobeen et al., 2018). Many factors can influence the composition and functions of intestinal microbiota, including dietary patterns, antibiotics, and the mode of delivery at birth, which plays an essential role in bacterial diversity (Kahleova et al., 2020; Tonon et al., 2021; Liou et al., 2019). Accumulating evidence indicates that perturbations in the gut microbiome and its influence on metabolic and physiological functions may significantly contribute to the development of human diseases. A reduction in the relative abundance of beneficial microbiota and an increase in pathogenic bacteria can disrupt the homeostasis of intestinal microbial flora composition and ecosystem, consequently influencing the body’s immune function and promoting the development of chronic inflammatory diseases (Stecher, 2015; Zhao et al., 2023). Psoriasis is also associated with gut microbiota. Numerous studies linked gastrointestinal health to skin homeostasis, revealing that both the composition and function of the intestinal microbial flora are disrupted in psoriasis patients. Some scholars have even suggested that psoriasis should be classified as a gastrointestinal disease (Ely, 2018).

*Tripterygium hypoglaucum* (Lévêque) Hutch. (THH), a member of the Celastraceae family, is predominantly found in southwestern China and along the southern bank of the Yangtze River. Its primary chemical constituents include alkaloids, diterpenoids, and triterpenoids (Zhang et al., 2016). Clinically, its pharmacological effects, such as antitumor, immunosuppressive, and anti-inflammatory activities, are comparable to those of Tripterygium wilfordii Hook F (TwHF) (Liu, 2011; Lu et al., 2015) but with fewer side effects and lower toxicity (Zhao et al., 2021).

Our preliminary studies have demonstrated that THH-MeOH effectively inhibits M5-induced hyperproliferation of HaCaT cells, reduces skin lesions and serum inflammatory cytokine levels along with their mRNA expression, corrects the IMQ-induced imbalance in the RORγt/Foxp3 ratio, and suppresses the phosphorylation of IκBα, NF-κB p65, MAPK, and STAT3/JAK2 (Sun et al., 2024). Oral administration of THH inhibits NOD-like receptor pyroptosis-related protein 3 (NLRP3)-mediated pyroptosis in arthritis mice, thereby blocking inflammatory responses in bilateral joints and the colon. It also significantly reshapes bile acid metabolism pattern in rheumatoid arthritis (RA) mice, demonstrating notable regulatory effects on intestinal microbial flora structure and metabolic status (Zheng et al., 2023). Current research on the gut microbiota-mediated metabolic regulation mechanisms of THH in psoriasis treatment remains limited, highlighting the need for comprehensive studies to elucidate its metabolic pathways. Building upon this research foundation, our study systematically investigates the therapeutic mechanisms of THH in IMQ-induced psoriasis in mice using a combination of metabolomics and 16S rRNA sequencing of gut microbiota. By establishing fecal microbiota transplantation (FMT) mouse models and conducting co-housing experiments, we identified THH-associated metabolic pathways and functional intestinal microbial flora that improve psoriasis while observing microbial composition and metabolite changes across different treatment groups.

## 2 Methods and materials

### 2.1 Extraction and quality control of THH extract

THH was extracted using methanol according to the established protocol of the project team. Our preliminary research indicated that a dose of 500 mg/kg/day of THH is the most effective for treating psoriasis. Therefore, this study also administered the same dose *via* oral gavage (Sun et al., 2024). The THH were collected from Wafang Township, Longyang District, Baoshan City, Yunnan Province, China, on 3 November 2020. The plant material was authenticated by Kunming Caizhi Biotechnology Co. A voucher specimen (No. 20221103) was deposited in the Key Laboratory of Chemistry in Ethnic Medicinal Resources, Yunnan Minzu University, Kunming, China. The dried rhizomes were pulverized and extracted four times with 95% methanol *via* maceration. The combined extracts were filtered and concentrated under reduced pressure to obtain the crude THH methanol extract (THH-MeOH). The drug-extract ratio (DER) was 10:1 (10 kg of dried rhizomes yielded 1.1 kg of dried extract).

#### 2.1.1 HPLC fingerprint and quantitative analysis

HPLC analysis was performed on an Agilent 1260 Infinity system equipped with a DAD detector. The separation was achieved on an Agilent ZORBAX SB-C18 column (4.6 × 250 mm, 5 μm) at 25 °C. The mobile phase consisted of 0.05% phosphoric acid water (A) and acetonitrile (B) with gradient elution: 0–13 min: 25% B; 13–73 min: 25% → 90% B; 73–93 min: 90% → 100% B; The detection wavelength was 214 nm, flow rate was 1.0 mL/min, and injection volume was 10 μL. The quantities of three marker compounds were determined as follows: triptolide, 0.79‰; celastrol, 0.46‰; and 3-O-acetyloleanolic acid, 0.76‰.

#### 2.1.2 LC-MS fingerprint

LC-MS analysis was performed on a 6240 Triple Quad LC/MS (Agilent, United States) with an ESI source. The chromatographic conditions were consistent with HPLC. Mass spectra were recorded in positive ion mode with a scan range of m/z 100–1000. The total ion chromatogram (TIC) was used as the LC-MS fingerprint.

#### 2.1.3 TLC fingerprint

TLC was performed on silica gel GF254 plates. The developing solvent system was petroleum ether–ethyl acetate at an optimized ratio. The spots were visualized under UV light at 254 nm and by heating the plates after spraying with a 10% sulfuric acid solution in ethanol.

### 2.2 Experimental animals and experimental design

**FIGURE 1:**
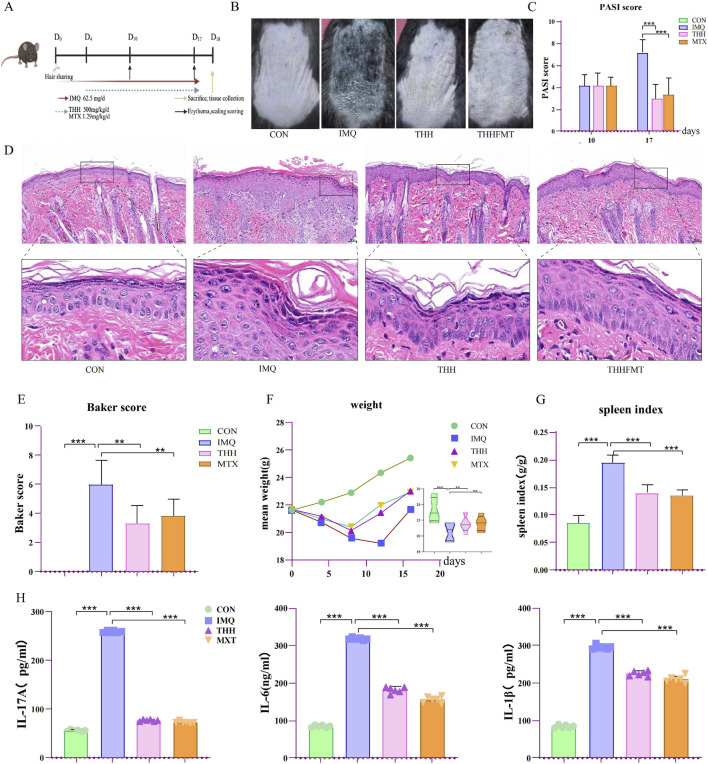
The effects of THH on IMQ-induced psoriasiform mouse models. **(A)** Experimental design flowchart; **(B)** Representative images of skin lesions in mice; **(C)** Comparison of PASI scores at the experimental endpoint; **(D)** Histopathological analysis of skin tissue; **(E)** Comparison of Baker scores among groups; **(F)** Dynamic changes in body weight during the experimental period; **(G)** Statistical analysis of spleen index (g/g) in each group; **(H)** Comparison of serum inflammatory factor levels among groups.

Eight-week-old specific pathogen-free male C57BL/6J mice were purchased from SPF (Beijing) Biotechnology Co., Ltd. (Beijing, China), weighing (20 ± 2) g. They were housed in the barrier environment of the Center for Experimental Animals of the Yunnan Hospital of Traditional Chinese Medicine (SYXK (Dian) K2022-0007), which was maintained at a relative humidity of 40%–70%, a temperature of 22 °C ± 2 °C, and a 12-h light/dark cycle, with free access to sterile water and commercial chow during the adaptive feeding period. Animal experiments were conducted in accordance with the National Guidelines for Experimental Animal Welfare and were approved by The Animal Ethics and Welfare Committee of Yunnan Hospital of Traditional Chinese Medicine (DW-2024-059). Every effort was made to minimize the number of animals used and to reduce animal suffering. Psoriasis in mice was induced following a previously described method (Lu et al., 2021). The experimental protocols are illustrated in Figure 1A. Specifically, mice were randomly divided into six groups (n = 6): control (CON), IMQ, THH, THHFMT, methotrexate (MTX), and co-housing (psoriatic mice with THH mice). An amount of 62.5 mg of 5% imiquimod (Sichuan MingXin Pharmaceutical Co., LTD., H20030129) was evenly applied to the dorsal skin after shaving daily for 14 days to induce psoriasis in the mice. Mice in the MTX group were gavaged with 1.29 mg/kg/day of methotrexate (SPH Sine Pharmaceutical Laboratories, Shanghai, China) dissolved in 0.9% saline; 0.85% saline was administered to the mice in the control and IMQ groups; and 500 mg/kg/day of THH was orally administered to the mice in the THH group correspondingly (Figure 1A).

### 2.3 Fecal preparation and fecal microbial transplantation

**FIGURE 2:**
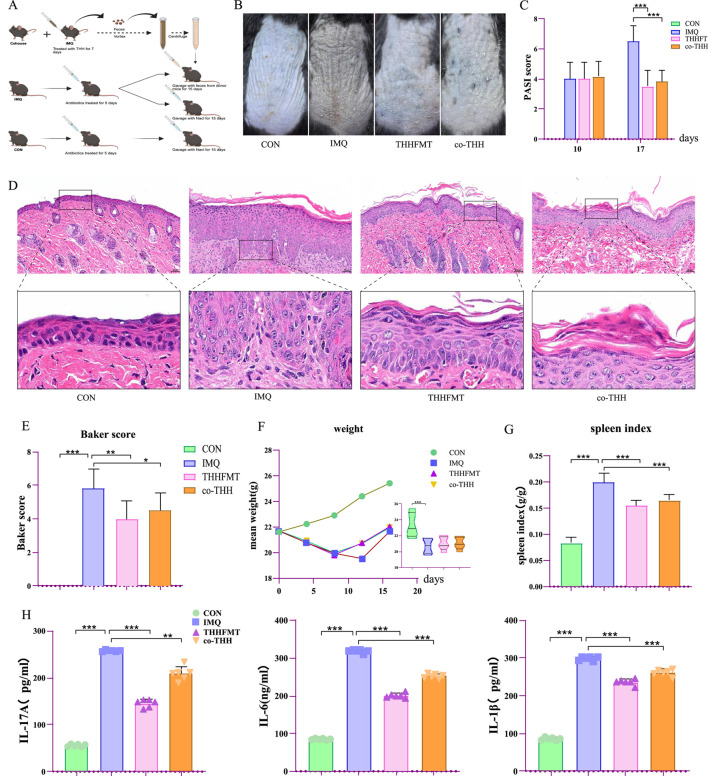
The therapeutic effects of THH-FMT and co-THH on IMQ-induced psoriasiform mice. **(A)** Experimental design flowchart; **(B)** Representative images of skin lesions in mice; **(C)** Comparative analysis of PASI scores; **(D)** Histopathological analysis of skin tissue; **(E)** Comparison of Baker scores among groups; **(F)** Dynamic changes in body weight during the experimental period; **(G)** Statistical analysis of spleen index (g/g) in each group; **(H)** Comparison of serum inflammatory factor levels among groups.

Before FMT and co-housing, mice were administered an antibiotic cocktail to deplete the intestinal microbiota, a method proven to effectively reduce intestinal microbes in mice (Shao-Yu et al., 2025). The treatment lasted for 5 days, during which each mouse received 200 μL of an antibiotic cocktail (administered by oral gavage following a 6-h fast). This cocktail contained 1 g/L ampicillin, 0.5 g/L neomycin, 0.5 g/L vancomycin, and 1 g/L metronidazole. A sterile reduced: 0.5 g/L-cysteine solution and 0.2 g/L Na2S were prepared by dissolving in PBS buffer, followed by filtrating through a 0.22 μm filter head for sterilization. After THH gavage treatment, feces were collected from therapeutic group mice (100 μg/10g) into 15 mL BD tubes. Three milliliters of sterile reduced PBS were added to the collected feces, and the mixture was stirred with a 1 mL sterile syringe until turbid. The turbid mixture was then filtered through a 70 μm sieve or centrifuged at 500 *g* for 3 min to obtain the supernatant, discarding the fecal residue. A volume of 100 μL of supernatant per 10 g of body weight was administered to each FMT mouse *via* gavage, 2-3 times weekly for two consecutive weeks (Figure 2A). Intestinal content samples were collected at the end of the FMT period and stored at −80 °C for 16S rRNA gene sequencing.

### 2.4 Co-housing

Six IMQ-mice were co-housed with six THH mice per cage. After 4 weeks of co-housing (Figure 2A), the mice were sacrificed to harvest epidermis and spleen tissue. Blood samples were collected at the end of the co-housing period and stored at −80 °C for *post hoc* tests.

### 2.5 PASI, body weight and spleen index

An objective scoring system was employed based on the clinical psoriasis area and severity index (PASI). Erythema, scaling, and thickening were assessed independently on a scale from 0 to 4, where 0 indicated none, 1 slight, 2 moderate, 3 marked, and 4 very marked. The level of erythema was evaluated using a scoring table with red taints. The cumulative score, which included erythema (0-4), scaling (0-4), and thickening (0-4), served as a measure of the severity of inflammation, with a total scale ranging from 0 to 12. Two scorers, who were blinded to the experimental groups, assessed the mice, and the average PASI scores were calculated. Mice were weighed once daily; following a 12-h fasting period, their fasting body weight was recorded. Subsequently, the mice were sacrificed to collect the spleen, which was rinsed with pre-cooled normal saline. Excess water was absorbed with filter paper, and the wet weight of the spleen was obtained. The spleen index was calculated using the following formula: Spleen index = spleen wet weight (g)/body weight (g).

### 2.6 Histological observation of murine skin lesions

Epidermis and spleen tissues were fixed in 4% paraformaldehyde in PBS, embedded in paraffin, sectioned, and stained with H&E for histopathological examination. Images were captured using an Olympus BX600 microscope. A pathological score based on the Baker score system was obtained to assess the severity of psoriasiform skin inflammation in epidermal tissues.

### 2.7 Serum cytokine measurements

Serum samples were collected and stored at −80 °C until analysis. After thawing, cytokine levels in the mouse plasma were measured. All ELISA kits were purchased from Bioswam Company, including those for IL-1β (Cat. no.: MU30369), IL-6 (Cat. no.: MU30044), and IL-17A (Cat. no.: MU30074). All procedures were conducted in accordance with the instructions provided with the kits. Finally, absorbance values were detected using a microplate reader, and the concentrations of the cytokines were determined based on standard curves.

### 2.8 16S RNA gene sequencing analysis of gut microbiota

Genomic DNA was extracted from intestinal contents samples using the CTAB/SDS method. After passing quality control, the DNA was diluted and subjected to PCR amplification with barcode-labeled universal primers targeting the V4 region (515F/806R), utilizing Phusion® High-Fidelity PCR Master Mix with GC Buffer. The amplified products were purified using a magnetic bead-based protocol, and target fragments were recovered with the Qiagen Gel Extraction Kit. Library construction was performed using the TruSeq® DNA PCR-Free Library Preparation Kit. Qualified libraries, verified through quality inspection, were sequenced on the Illumina NovaSeq 6000 platform with 250-bp paired-end (PE250) sequencing. Raw data were quality-filtered using fastp to obtain high-quality paired-end reads, which were then assembled into Clean Tags using FLASH (v1.2.11). Chimeric sequences were removed by alignment against the SILVA database (v138.1) using vsearch (v2.27.1) to generate effective tags. Subsequently, operational taxonomic units (OTUs) were clustered using the Uparse algorithm with a 97% sequence similarity cutoff, and amplicon sequence variants (ASVs) were generated *via* the Deblur/DADA2 pipeline in QIIME 2. Taxonomic annotation was conducted using mothur through alignment against the SILVA database, covering taxonomic levels from phylum to species. After normalizing the annotation results, α-diversity indices (including Chao1, ACE, Shannon, and Simpson evenness indices) were calculated, and OTU rank abundance curves along with α-diversity rarefaction curves were plotted. Unifrac distances were computed using the R package phyloseq to construct UPGMA sample clustering trees. Meanwhile, PCA, Principal Component Analysis (PCA), Principal Coordinate Analysis (PCoA), and Non-metric Multidimensional Scaling (NMDS) plots were generated for beta-diversity analysis. Linear Discriminant Analysis Effect Size (LEfSe) was employed to identify differentially abundant taxa among groups, using a threshold Linear Discriminant Analysis (LDA) Score of 4.

### 2.9 Analysis of intestinal contents metabonomics

Pretreatment and analysis of intestinal contents samples were conducted by MetWare Biotechnology Co., Ltd. (Wuhan, China) using a liquid chromatography-mass spectrometry (LC-MS) platform. Chromatographic separation was achieved with a Waters ACQUITY Premier HSS T3 column (1.8 µm, 2.1 mm × 100 mm), and the column temperature was maintained at 40 °C. For the mobile phase in positive ion mode, 0.1% formic acid in water (phase A) was used alongside acetonitrile (phase B); in negative ion mode, 10 mM ammonium formate in water (phase A) was paired with acetonitrile (phase B). The gradient elution program was as follows: 0–1 min, 2% B; 1–3 min, 2%→20% B; 3–9 min, 20%→60% B; 9–15 min, 60%→99% B; 15–23.5 min, 99% B; 23.5–31.1 min, 99%→5% B; 31.1–40.1 min, 5% B. The flow rate was set at 0.4 mL/min, and the injection volume was 4 μL. Raw mass spectrometric data were converted to mzML format using ProteoWizard, followed by peak extraction, peak alignment, and retention time correction *via* XCMS. After normalizing the total peak intensity, multivariate statistical analysis was performed using the ropls R package (v1.6.2): PCA was employed to evaluate inter- and intra-group sample differences; heatmaps were generated with the ComplexHeatmap R package after unit variance (UV) scaling to conduct hierarchical cluster analysis (HCA) of metabolite accumulation patterns; Orthogonal Partial Least Squares-Discriminant Analysis (OPLS-DA) was utilized to explore metabolic profile differences among groups; and one-way analysis of variance (ANOVA) was applied to test the significance of metabolite content differences between groups. Differential metabolites were screened based on the criteria: Log_2_FC > 3, VIP >1.0, p < 0.05. Volcano plots were created to visualize the effects of THH and THHFMT treatments on potential biomarkers of intestinal contents metabolites in IMQ-induced psoriasis mice.

### 2.10 Pathway analysis

For microbial samples, the functional composition was inferred using PICRUSt2 software based on the taxonomic composition derived from 16S rRNA gene sequencing data, enabling the analysis of functional differences among various samples or groups (Douglas et al., 2020). The specific steps were as follows: First, to account for variations in 16S rRNA gene copy numbers across different species, normalization was applied to the generated OTU table. Subsequently, KEGG family annotation information was obtained by matching the GreenGene ID corresponding to each OTU. The abundance of KEGG functional families was further calculated, and relevant metabolic pathways were extracted from the KEGG database. For metabolite functional analysis, the differential metabolites identified from intestinal contents metabolomics were imported into the MetaboAnalyst 4.0 online database (https://www.metaboanalyst.ca/) for pathway enrichment analysis. This database facilitates statistical analysis, functional annotation, and integrated analysis of metabolomic data. Prior to functional analysis, it automatically maps compound names to identifiers in multiple databases, including KEGG, HMDB, MSEA, ChEBI, METLIN, and PubChem. Metabolic pathways with *P* < 0.05 were selected as potential functional pathways through which THH affects IMQ-induced psoriasis in mice.

### 2.11 Correlation profiling

Pearson correlation analysis was conducted between altered metabolites identified from intestinal contents metabonomics and perturbed gut microbial genera identified from 16S rRNA gene sequencing analysis. Additionally, correlation analyses were performed between phenotypes and gut microbial genera as well as intestinal contents metabonomics. The criteria for screening included a correlation coefficient |r| > 0.70 and P < 0.05. Finally, significant correlations between metabolites and gut microbial genera were identified and presented as heat maps (^*∗*^ *P* < 0.05, ^*∗∗*^ *P* < 0.01). Red indicates a positive correlation, while blue indicates a negative correlation.

### 2.12 Statistical analysis

The data were presented as the mean ± SD. Biochemical data were analyzed using SPSS 26.0. Comparisons between two groups were performed using an unpaired, two-tailed Student’s t-test, whereas differences among multiple groups were evaluated using ANOVA. GraphPad Prism 8.0 was employed for plotting. The results were expressed as mean ± SD. Groups marked with different lowercase letters were considered significantly different (*P* < 0.05). *P* < 0.05 and *P* < 0.01 were regarded as significant and highly significant, respectively.

## 3 Results

### 3.1 THH ameliorated skin damage in IMQ-induced psoriasis like mice

Both THH and MTX treatments exerted protective effects to varying degrees on IMQ-induced psoriasis in mice (Figure 1B). Compared to the IMQ group, the PASI score was reduced in the THH group (P = 0.000), demonstrating slightly superior efficacy relative to the MTX group (Figure 1C). HE staining of skin tissues revealed that the CON group exhibited an intact epidermis, a thin stratum corneum, and no infiltration of inflammatory cells. In contrast, the IMQ group displayed marked epidermal hyperplasia, desquamation of the stratum corneum, and extensive inflammatory cell infiltration. Both THH and MTX treatments mitigated acanthosis (Figure 1D). Histopathological scores indicated that the scores for the THH(P = 0.001) and MTX groups (P = 0.005) were significantly lower than those of the IMQ group (Figure 1E). IMQ treatment resulted in a significant decrease in body weight in mice from day 5 onwards (P = 0.001), whereas THH and MTX alleviated this trend, maintaining body weight above 20 g (Figure 1F). Additionally, the spleen index for the THH and MTX groups was lower than that of the IMQ group, with THH significantly reducing spleen weight (P = 0.000), Figure 1G). An increase in spleen weight suggests the release of inflammatory mediators, such as cytokines. The body weight of mice with psoriasis decreases due to underlying pathological conditions and can be restored following treatment. Therefore, an increase in spleen weight is regarded as a marker of immune cell accumulation within the spleen, whereas an increase in body weight indicates a return to baseline levels (Sulthana et al., 2023).

Both THHFMT and co-THH exhibited comparable therapeutic effects on IMQ-induced psoriasis in mice (Figure 2B). In comparison to the IMQ group, the PASI score in the THHFMT group was significantly reduced, demonstrating efficacy comparable to that of MTX (P = 0.000). The co-THH group alleviated skin lesions; however, no statistically significant difference was observed (Figure 2C). HE staining results indicated that THHFMT significantly attenuated acanthosis, while the degree of epidermal hyperplasia in the co-THH group was also mitigated compared to the IMQ group (Figure 2D). Histopathological evaluation revealed that the Baker score in the THHFMT group was significantly decreased (P = 0.003), whereas the score in the co-THH group showed no significant change (P = 0.376) (Figure 2E). Although neither THHFMT nor co-THH alleviated IMQ-induced weight loss (P = 0.000) (Figure 2F), both effectively reduced the spleen weight of the mice (P = 0.000) (Figure 2G). Additionally, THH, THHFMT, and co-THH demonstrated varying degrees of inhibitory effects on IL-1β, IL-6, and IL-17A in psoriasis mice (Figures 1H, 2H). FMT can directly reduce skin lesion scores, minimize epidermal thickening, and lower inflammatory cytokines such as IL-17 and IL-23, demonstrating that gut microbiota can independently influence anti-psoriasis therapeutic efficacy.

### 3.2 Sequencing data quality assessment and alpha diversity analysis

To verify the sufficiency of sequencing volume in covering the original microbial diversity, Alpha diversity analysis was performed based on 16S rRNA sequencing results with 97% similarity to assess the richness and evenness of intestinal flora within groups. The analyzed indices included Ace, Chao1, Observed ASV, Shannon, Simpson indices, and PD-whole-tree (where Chao1, Observed ASV, and Ace indices measure species richness; Shannon and Simpson indices evaluate species diversity; PD-whole-tree reflects species richness, diversity, and phylogenetic information simultaneously). Results revealed that the Observed ASV, Chao1, and Ace indices were significantly elevated in the CON group (Observed ASV ≈330, Chao1/Ace ≈350), decreased sharply in the IMQ group (Chao1, Observed ASV ≈ 270; Ace ≈2 80), and showed partial recovery in the THH and THHFMT groups (Supplementary Figure S1). This suggested that IMQ-induced psoriasis could lead to the loss of intestinal flora species in mice, and THH treatment, along with corresponding FMT, could regulate flora richness. Compared with the CON group, the Shannon and Simpson indices decreased in the IMQ group but recovered after THH treatment, with the THHFMT group showing the optimal recovery of flora evenness, indicating that both interventions could regulate flora diversity. Additionally, PD-whole-tree recovered after treatment, demonstrating that THH and THHFMT could promote the comprehensive restoration of the structure and function of the intestinal flora ecosystem in psoriasis mice.

### 3.3 Beta diversity analysis

**FIGURE 3:**
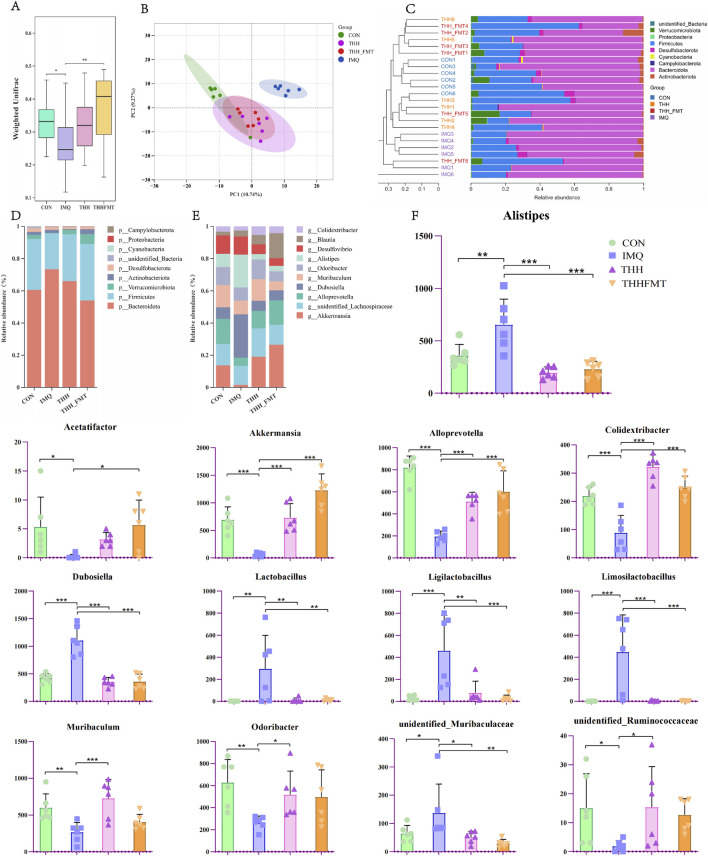
The regulation of THH and THH-FMT on intestinal microbial flora composition in IMQ-induced psoriasiform mice. **(A)** Beta diversity reflects the differences in microbial community structure among groups; **(B)** PCA plot of gut microbiota; **(C)** Hierarchical clustering analysis of UPGMA among groups; **(D)** Stacked bar chart showing the relative abundance of intestinal microbial flora at the phylum level; **(E)** Stacked bar plot of microbial community composition at the genus level across groups; **(F)** ANOVA analysis of gut bacteria at the genus level. Denotes comparisons with the IMQ group, ^*^ *P* < 0.05, ^**^ *P* < 0.01. ^***^ *P* < 0.001.

Beta diversity reflects the differences in microbial community structure among groups. The Adonis test revealed significant inter-group differences (Figure 3A). PCA indicated that the intestinal microbiota composition of the IMQ group was distinctly separated from that of the CON, THH, and THHFMT groups (Figure 3B). This inter-group difference was further confirmed by PCoA and NMDS analysis (Supplementary Figures S2A,B). Utilizing the Bray-Curtis distance matrix derived from the Beta diversity analysis, hierarchical clustering was conducted using the Unweighted Pair Group Method with Arithmetic Mean (UPGMA) *via* R software to assess the similarity of species composition among samples. A shorter distance and branch length between samples indicated a higher similarity in species composition. The results demonstrated that samples in the CON group clustered distinctly and were markedly separated from the IMQ group. Although samples in the THH and THHFMT groups were relatively scattered, they exhibited a high similarity in species composition (Figure 3C). The alteration in β-diversity induced by IMQ indicates that the gut microbiota in IMQ-induced psoriasis differs from that in a healthy state (Kim et al., 2025). Such gut microbiota may exacerbate skin inflammation by promoting Th17 responses (Kim et al., 2025) and disrupting the gut barrier (Yu et al., 2025). These changes in the microbiota are both a crucial aspect of the disease and a biological indicator for evaluating treatment efficacy.

### 3.4 Species annotation analysis and screening of significantly altered microbiota

Figures 3D,E illustrate the taxonomic annotation results of intestinal microbial flora at the phylum and genus levels, as revealed by 16S rRNA sequencing, presenting only the top 10 bacterial taxa with the highest relative abundance. At the phylum level, *Firmicutes* and *Bacteroidota* emerged as the two dominant phyla in the mouse gut microbiota, accounting for the highest proportion and demonstrating relative superiority across all samples. Compared to the CON group, the IMQ group exhibited a significant decrease in *Firmicutes*, an increase in *Bacteroidota*, and a reduced *Firmicutes/Bacteroidota* (F/B) ratio; this alteration serves as a marker of intestinal barrier impairment, consistent with the characteristic F/B ratio inversion observed in psoriasis patients (Wen et al., 2023). Previous studies have indicated that the imbalance between *Firmicutes* and *Bacteroidota* is primarily associated with energy metabolism disorders (Lin et al., 2018). At the genus level, in comparison to the CON group, the IMQ group displayed a significant decrease in *Odoribacter(P = 0.005)*, *Muribaculum(P = 0.004)*, *Alloprevotella(P = 0.000)*, *Acetatifactor(P = 0.017), Colidextribacter(P = 0.000), Akkermansia(P = 0.000),* and *unidentified* Ruminococcaceae*(P = 0.028)*. Conversely, the IMQ group displayed a significant increase in *Alistipes(P = 0.002)*, *unidentified Muribaculaceae(P = 0.032)*, *Dubosiella (P = 0.000)*, *Lactobacillus(P = 0.003)*, *Ligilactobacillus(P = 0.000)*, and Limosi*lactobacillus (P = 0.000)* (Figure 3F). Specifically, *Odoribacter*, *Muribaculum*, *Alloprevotella*, *Acetatifactor*, and *unidentified* Ruminococcaceae can ferment substrates such as dietary fiber to produce short-chain fatty acids (SCFAs) including acetate, propionate, and butyrate, and participate in the decomposition and utilization of complex carbohydrates (Fouad et al., 2022; Thio, 2018). *Odoribacter* is capable of producing SCFAs (Li et al., 2025), while metabolites such as acetate produced by *Acetatifactor* can influence keratinocyte proliferation, which is associated with elevated levels of IL-22, a cytokine that drives epidermal hyperplasia (Fouad et al., 2022). *Colidextribacter* is positively correlated with various SCFAs (e.g., acetate, propionate, isobutyrate, isovalerate), suggesting its potential involvement in the production or metabolic regulation of SCFAs. SCFAs play crucial roles in maintaining intestinal barrier function, exerting anti-inflammatory effects, and regulating energy metabolism (Xin et al., 2025; Wang Q. et al., 2022; Gao et al., 2024). *Akkermansia* is closely related to mucosal barrier function; its reduced abundance in psoriasis patients may exacerbate intestinal permeability and affect regulatory T (Treg) cell function by modulating SCFA production (Wen et al., 2023). *Alistipes* may contribute to the progression of psoriasis by interfering with bile acid metabolism, disrupting amino acid metabolism, and increasing the levels of psoriasis-related pro-inflammatory cytokines (Fu et al., 2024). *Dubosiella* can disrupt branched-chain amino acid (BCAA) metabolism, leading to BCAA accumulation in mouse colonic organoids. This mechanism may be associated with the progression of psoriasis complicated by obesity, as abnormal elevation of BCAA levels is often observed in obese psoriasis mice (Wang et al., 2024). Following THH and THHFMT treatments, the aforementioned differential genera were reversed: *Firmicutes* significantly increased (*P* < 0.05), *Bacteroidota* decreased, and the F/B ratio was normalized. No significant changes were observed in other phyla or genera, indicating that monotherapy has limited regulatory effects on the core gut phyla.

### 3.5 Predictive analysis of faecal microbial function and biomarker screening

**FIGURE 4:**
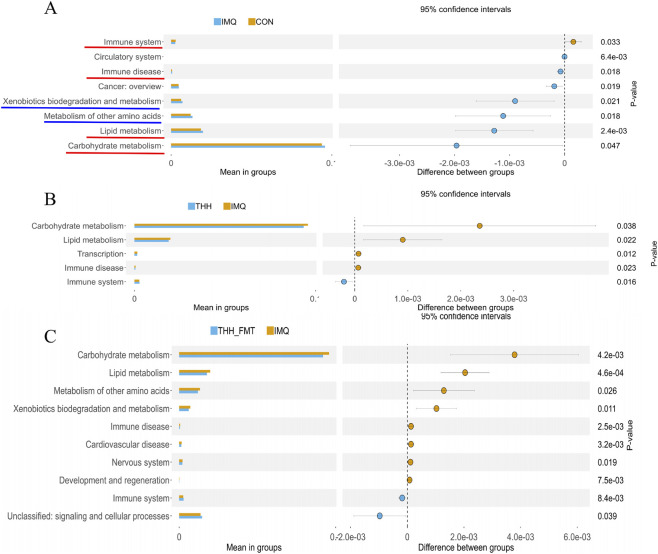
The KEGG functional prediction analysis of intestinal microbiota. **(A)** The enrichment analysis of differential functional pathways between the CON and IMQ groups; **(B)** The enrichment analysis of differential functional pathways between the THH and IMQ groups; **(C)** The enrichment analysis of differential functional pathways between the THH-FMT and IMQ groups. The red horizontal line indicates the differences between THH, THH-FMT compared to IMQ, while the blue horizontal line indicates that THH-FMT exhibits distinct characteristics.

KEGG pathway enrichment analysis revealed that the significantly enhanced pathways in psoriasis mice included the circulatory system, xenobiotics biodegradation and metabolism, metabolism of other amino acids, lipid metabolism, and carbohydrate metabolism, while the immune system pathway was significantly attenuated (Figure 4A). These PICRUSt2 analysis predicted that intestinal microbial flora dysregulation, which leads to impaired immune system function and abnormal enhancement of related metabolic pathways, may play a crucial role in the pathogenesis of PSO. Additionally, after THH and THHFMT treatments, pathways associated with THH were identified, including the immune system, immune disease, xenobiotics biodegradation and metabolism, metabolism of other amino acids, lipid metabolism, and carbohydrate metabolism. The color-labeled pathways in Figure 4A represent potential regulatory pathways linked to THH and THHFMT mediated changes in gut microbiota.

### 3.6 Intestinal contents metabolic pattern of THH in treating psoriasis-like mice

**FIGURE 5:**
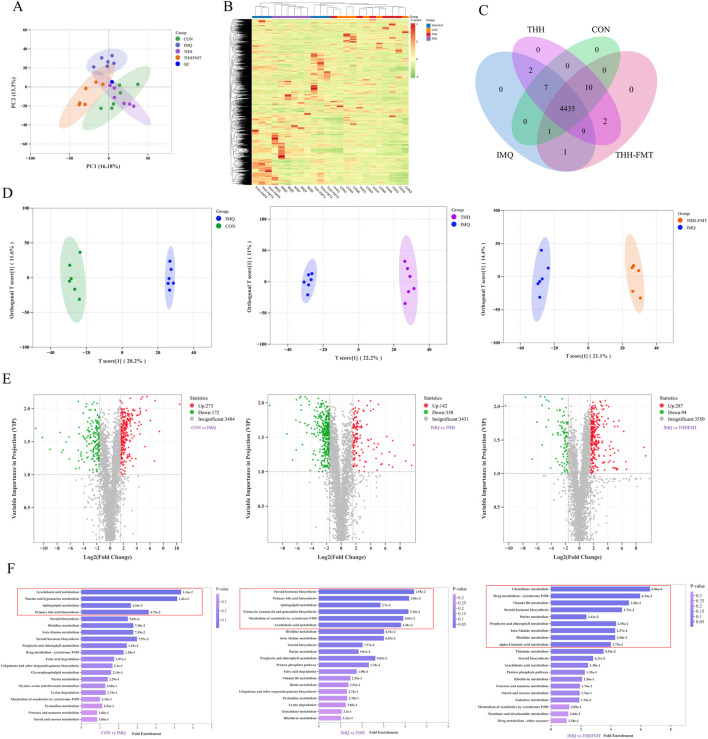
The effects of THH and THH-FMT on intestinal content metabolites in IMQ-induced psoriasiform mice. **(A)** The PCA score plot among groups; **(B)** The heatmap showing hierarchical clustering of differential intestinal content metabolite relative abundances among groups; **(C)** The Venn diagram of intestinal content metabolites across the CON, IMQ, THH, and THH-FMT groups; **(D)** The OPLS-DA score plot of intestinal content metabolites among the CON, IMQ, THH, and THH-FMT groups; **(E)** VIP scatter plots of intestinal content metabolites in pairwise comparisons; **(F)** showcases metabolic pathway enrichment bar plots of intestinal metabolites among groups.

PCA score plots were utilized to analyze the principal components of intestinal contents metabolomes across the four groups. The results indicated that the principal components effectively distinguished the four groups: the CON, THH, and THHFMT groups were clearly separated from the IMQ group, and the metabolomic similarity between the THH/THHFMT groups and the CON group was significantly higher than that between the THH/THHFMT groups and the IMQ group (Figure 5A). Hierarchical clustering heatmaps and Venn diagrams further illustrated the global differences in metabolic characteristics among the groups (Figures 5B,C). Furthermore, partial least squares discriminant analysis (PLS-DA), which possesses stronger discriminative power, was employed for metabolomic classification. The PLS-DA model demonstrated a significant clustering effect, confirming notable metabolic differences between the IMQ group and the CON/THH/THHFMT groups (Figure 5D and Supplementary Figure S3 score plots). Differential metabolites were screened based on the criteria: VIP >1, Log_2_ fold change (FC) > 3, and *P* < 0.05. Specifically, compared to the CON group, the IMQ group exhibited 273 upregulated and 172 downregulated metabolites; in comparison to the IMQ group, the THH group showed 142 upregulated and 358 downregulated metabolites, while the THHFMT group had 287 upregulated and 94 downregulated metabolites (Figure 5E). For the differential metabolites identified in each group comparison, the top 10 metabolites with the highest VIP values in the OPLS-DA model are presented in Supplementary Figure S3B. Following qualitative and quantitative analysis of all detected metabolites, the fold change differences among groups were compared, and Supplementary Figure S3C displays the top 20 metabolites with the largest fold changes in each group comparison. These results suggest that THH-reshaped microbiota may contribute to the anti-psoriatic effect, although the absence of a vehicle-FMT control (healthy donor microbiota) limits the specificity of this conclusion.

### 3.7 Key metabolic pathway analysis for different metabolites from feces

IMQ-induced psoriatic mice exhibited systemic metabolic hyperactivity (Figure 5F), with core differential metabolic biomarkers enriched in the following pathways: arachidonic acid metabolism, taurine and hypotaurine metabolism, sphingolipid metabolism, and primary bile acid biosynthesis. Specifically, arachidonic acid, an antecedent of inflammatory mediators such as prostaglandins and leukotrienes, is metabolically activated by cyclooxygenase to generate abundant pro-inflammatory factors, which directly exacerbate skin erythema, scaling, and pruritus in psoriasis, serving as a signature inflammation-driven pathway (Morin et al., 2023). Sphingolipids are precursors of key components (e.g., ceramides) in the skin’s stratum corneum barrier; dysregulation of this pathway impairs skin barrier integrity, facilitating the invasion of external stimuli and reversely activating immune-inflammatory responses (Bochenska and Gabig-Ciminska, 2020). After THH intervention, differential metabolites were primarily enriched in steroid hormone biosynthesis, primary bile acid biosynthesis, sphingolipid metabolism, neomycin, kanamycin, and gentamicin biosynthesis, metabolism of xenobiotics by cytochrome P450, and arachidonic acid metabolism. In contrast, differential metabolites following THHFMT intervention were concentrated in glutathione metabolism, drug metabolism *via* cytochrome P450, vitamin B6 metabolism, steroid hormone biosynthesis, purine metabolism, porphyrin and chlorophyll metabolism, beta-alanine metabolism, histidine metabolism, and alpha-linolenic acid metabolism.

Among the specific differential metabolites, the following compounds exhibited significant differences among groups (Supplementary Figures S4 and S5): those involved in the steroid hormone biosynthesis pathway include cholesterol, dehydroepiandrosterone, aldosterone, progesterone, 20α-hydroxycholesterol, tetrahydrocorticosterone, and allopregnanolone; those in the primary bile acid biosynthesis pathway include cholesterol, 25-hydroxycholesterol, glycine, taurine, and cholic acid; those in the sphingolipid metabolism pathway consist of sphingosine, sphingosine 1-phosphate, phytosphingosine, sphinganine 1-phosphate, L-serine, and 3-dehydrosphinganine; the key metabolite in the arachidonic acid metabolism pathway is leukotriene B4; those involved in vitamin B6 and amino acid metabolism include pyridoxine, pyridoxal, pyridoxamine, carnosine, uracil, and L-histidine; and those in the drug toxicity metabolism pathway comprise naphthalene, endoxifen, morphine-6-glucuronide, 1-nitro-5,6-dihydroxy-dihydronaphthalene, and glutathione.

### 3.8 Correlation analysis for differential metabolites and microbes

**FIGURE 6:**
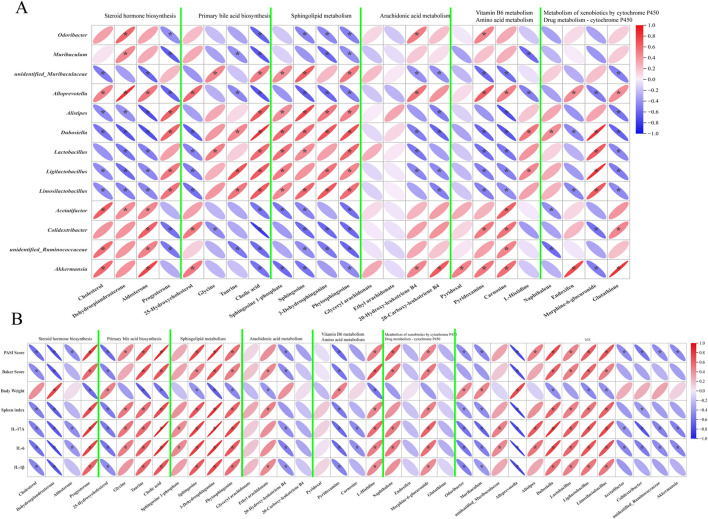
Composite correlation analyses of differential bacterial genera, intestinal content metabolites, and disease phenotypes. **(A)** The correlation analysis of differential bacterial genera and intestinal content metabolites, **(B)** The correlation analysis of differential bacterial genera, intestinal content metabolites, and psoriasis-related disease phenotypes.

To elucidate the functional associations among intestinal microbial flora dysbiosis, metabolic perturbations, and disease phenotypes in IMQ-induced psoriasis mice, Pearson correlation analysis was conducted to evaluate the relationships between 13 differential gut genera and 24 key intestinal contents differential metabolites. These metabolites are enriched in pathways such as steroid hormone biosynthesis, primary bile acid biosynthesis, sphingolipid metabolism, arachidonic acid metabolism, vitamin B6 metabolism, amino acid metabolism, metabolism of xenobiotics by cytochrome P450, and drug metabolism (Figure 6A).

The results indicated that steroid hormone biosynthesis was positively correlated with *Acetatifactor* and *Akkermansia*, while it exhibited a negative correlation with *unidentified* Ruminococcaceae. Both primary bile acid biosynthesis and sphingolipid metabolism showed positive correlations with *Alistipes*, *Dubosiella*, *Lactobacillus*, *Ligilactobacillus*, and Limosi*lactobacillus*, but negative correlations with *Odoribacter*, *Muribaculum*, *Alloprevotella*, *Acetatifactor*, *Colidextribacter*, *unidentified* Ruminococcaceae, and *Akkermansia*. In contrast, arachidonic acid metabolism was negatively correlated with *unidentified Muribaculaceae*, *Alistipes*, *Dubosiella*, *Lactobacillus*, *Ligilactobacillus*, and Limosi*lactobacillus*, while it was positively correlated with *Odoribacter*, *Alloprevotella*, and *Akkermansia*. Both vitamin B6 metabolism and amino acid metabolism were negatively correlated with *unidentified Muribaculaceae*, *Alistipes*, *Dubosiella*, *Lactobacillus*, *Ligilactobacillus*, and Limosi*lactobacillus*, and positively correlated with *Odoribacter*, *Alloprevotella*, *Acetatifactor*, *Colidextribacter*, *unidentified* Ruminococcaceae, and *Akkermansia*. The metabolism of xenobiotics by cytochrome P450 was positively correlated with *Dubosiella* and negatively correlated with *Acetatifactor* and *unidentified* Ruminococcaceae; drug metabolism *via* cytochrome P450 was positively correlated with *Alloprevotella* and *Akkermansia*, but negatively correlated with *Dubosiella* and Limosi*lactobacillus*.

Further analysis of the correlations between differential genera and disease phenotypes (Figure 6B) revealed that the PASI score, Baker score, spleen index, and levels of cytokines IL-17A, IL-6, and IL-1β were generally negatively correlated with *Odoribacter*, *Muribaculum*, *Alloprevotella*, *Acetatifactor*, *Colidextribacter*, *unidentified* Ruminococcaceae, *Akkermansia*, as well as metabolites related to steroid hormone biosynthesis and vitamin B6 metabolism. In contrast, these phenotypic indices were generally positively correlated with *unidentified Muribaculaceae*, *Alistipes*, *Dubosiella*, *Lactobacillus*, *Ligilactobacillus*, Limosi*lactobacillus*, and metabolites involved in primary bile acid biosynthesis, sphingolipid metabolism, amino acid metabolism, metabolism of xenobiotics by cytochrome P450, and drug metabolism, although some correlations did not reach statistical significance.

## 4 Discussion

The intestinal microbial flora is characterized by a diverse collection of microorganisms residing in the digestive systems of humans. Compared to other regions of the body, it contains the highest number of microorganisms and the greatest variety of species (Thursby and Juge, 2017). This collection primarily consists of bacteria; however, viruses and other eukaryotes begin to invade the gastrointestinal tract shortly after birth (Li et al., 2021; Kuang et al., 2016). The human intestinal microbial flora starts to develop during the perinatal stage and is essential for the normal functioning of the host organism (Forsgren et al., 2017). It can produce various metabolic products that interact with the host, which can have both positive and negative impacts on human health. Disruption in the production of SCFAs may lead to a range of pathogenic consequences for the host (Zhuang et al., 2019). Furthermore, the metabolic products of the gut microbiota, such as fatty acids, also play a significant role in maintaining intestinal mucosal health. Notably, levels of medium-chain fatty acids (MCFAs), including caprylic acid and capric acid, were found to be significantly lower in intestinal contents samples from patients with psoriatic arthritis and psoriasis compared to healthy individuals. The antimicrobial properties of MCFAs are vital for sustaining intestinal microbial flora homeostasis (Scher et al., 2015).

Numerous studies have demonstrated that psoriasis is associated with an imbalance in intestinal flora. Recent data confirm the presence of gut-derived bacterial DNA in the blood of psoriatic patients. The identified species include *Escherichia coli*, *Klebsiella pneumoniae*, *Enterococcus faecalis*, *Proteus mirabilis*, *Streptococcus* pyogenes, and *Shigella* flexneri (Ramirez-Bosca et al., 2015). This finding underscores the association between the gut and psoriasis. Psoriasis patients exhibit dysbiosis of gut microbiota, characterized by a significant increase in heterogeneity: the abundance of *Bacteroidota* is decreased, while *Firmicutes* and Actinobacteriota predominate. MD proposed that psoriasis could also be classified as a type of intestinal disease (Ely, 2018). Our IMQ-induced psoriasis model mice exhibited gut microbial dysbiosis, which was effectively reversed by THH and THH-FMT interventions. Pathway enrichment analysis revealed that the regulated genera primarily target immune-related pathways, xenobiotic metabolism, and lipid/amino acid metabolism, which are closely intertwined with the pathogenesis of psoriasis. Below, we focus on the molecular mechanisms underlying the therapeutic effects of THH, emphasizing the crosstalk between gut microbiota, metabolic pathways, and skin inflammation.

### 4.1 Sphingolipid metabolism

*Akkermansia*-mediated regulation of S1P signaling plays a crucial role in repairing the skin barrier. Sphingolipid metabolism is a core regulatory pathway in psoriasis, as its dysregulation directly impairs skin barrier function through reduced ceramide levels and promotes keratinocyte hyperproliferation and inflammation *via* elevated sphingosine-1-phosphate (S1P) (Cho et al., 2004; Nakajima et al., 2013), Mice with conditional knockout of serine palmitoyltransferase (SPT), the rate-limiting enzyme for sphingolipid synthesis, in keratinocytes (SPT-cKO) exhibit epidermal ceramide deficiency and typical psoriatic dermatitis phenotypes (Nakajima et al., 2013), directly verifying the causal link between sphingolipid depletion and barrier impairment. Furthermore, S1P, a key product of sphingolipid metabolism, inhibits keratinocyte apoptosis and promotes excessive proliferation by activating downstream signaling pathways of S1P receptors (S1PRs), such as the ERK1/2 and PI3K/Akt pathways (Masuda-Kuroki et al., 2023; M et al., 2017). Clinical studies have shown elevated serum S1P levels and decreased total ceramide content in psoriasis patients. This metabolic imbalance may further exacerbate abnormal hyperplasia of lesional tissues through S1PR2-mediated pro-proliferative effects (M et al., 2017; Kozlowska et al., 2019), forming a pathological cycle. Additionally, sphingolipid derivatives (e.g., ceramides, S1P) can directly regulate immune cell function and inflammatory factor release. Sustained activation of the S1P-S1PR signaling axis in psoriatic lesions promotes Th17 cell differentiation and IL-17 production, driving downstream inflammatory cascades (Masuda-Kuroki et al., 2023; Li et al., 2020). Notably, anti-TNF-α therapy can partially correct the abnormal expression of circulating sphingolipid profiles in patients, indicating a bidirectional regulatory relationship between sphingolipid metabolism and inflammatory pathways that collectively contribute to disease pathogenesis.

Gut microbiota-derived sphingolipids can be traced to the epidermal tissue *via* bioorthogonal labeling experiments. Mice colonized with sphingolipid synthesis-deficient bacterial strains exhibit significantly reduced levels of skin sphingolipids and abnormal expression of epidermal barrier-related genes (Lee et al., 2024). This alteration directly affects the substrate supply for host sphingolipid synthesis. Additionally, microbiota dysbiosis reduces the production of SCFAs, which are known to regulate skin inflammation *via* the FXR/NF-κB pathway (Wu et al., 2025) and inhibit IL-17 production by γδT cells, thereby indirectly impacting the inflammatory regulatory network associated with sphingolipid metabolism. Clinically significant associations exist between differences in intestinal microbial flora composition across various psoriasis subtypes and dysregulation of sphingolipid metabolism (Wang X. et al., 2022). Additionally, serum sphingolipid profile characteristics may serve as potential biomarkers for psoriatic arthritis (M et al., 2017). Furthermore, abnormalities in sphingolipid metabolism are more pronounced in obese psoriasis patients (Kozlowska et al., 2019; Mao et al., 2025), providing new insights for gut-targeted therapy. Fingolimod, which targets S1PR1/3/4/5, has been shown to exert anti-inflammatory effects and regulate keratinocyte proliferation in studies (Masuda-Kuroki et al., 2023).

Our study identified a strong correlation between sphingolipid metabolism and specific gut genera, with *Akkermansia* (a mucus-protecting bacterium) showing a negative correlation with pro-inflammatory sphingolipids. We propose that THH exerts its therapeutic effects by restoring *Akkermansia* abundance, which modulates sphingolipid metabolism through two key mechanisms, which inhibiting S1P production or promoting its degradation, thereby reducing S1PR2-mediated keratinocyte hyperproliferation; and alleviating intestinal oxidative stress (*via* synergistic regulation of glutathione metabolism, as indicated by THH-FMT-enriched glutathione pathways), which reduces ROS-induced damage to the intestinal barrier and indirectly improves epidermal ceramide synthesis. This *Akkermansia*-sphingolipid axis ultimately repairs the skin barrier and suppresses Th17-driven inflammation, consistent with clinical observations that anti-TNF-α therapy can reverse abnormal sphingolipid profiles in psoriasis patients.

### 4.2 Arachidonie acid metabolism

Microbiota-driven imbalance of pro- and anti-inflammatory mediators associated with arachidonic acid (AA) metabolism contributes to the pathogenesis of psoriasis by generating pro-inflammatory derivatives like leukotrienes and prostaglandins, which amplify both local and systemic inflammation (Mayser et al., 2002; Vahlquist et al., 1985). The imbalance between pro-inflammatory ω-6 polyunsaturated fatty acids (PUFAs), such as AA, and anti-inflammatory ω-3 PUFAs, along with dysregulated fatty acid desaturase 2 (FADS2) activity, further exacerbates this inflammatory response (Mayser et al., 2002; Cai et al., 2025). Intestinal microbial flora modulates AA metabolism primarily through the production of short-chain fatty acids (SCFAs), such as butyrate, which inhibit NF-κB and STAT3 pathways to suppress AA-mediated inflammation (Paine et al., 2023; Simancas-Racines et al., 2025). Conversely, microbiota-induced intestinal barrier disruption promotes lipopolysaccharide translocation and activates AA metabolism in immune cells (Hong et al., 2025). Our results indicate that THH and THH-FMT reverse AA metabolic dysregulation in psoriasis mice, closely associated with the restoration of SCFA-producing bacteria and the reduction of pro-inflammatory genera, such as Alistipes. Specifically, the recovery of butyrate-producing bacteria following intervention likely inhibits AA release from cell membranes and suppresses the lipoxygenase pathway, thereby reducing pro-inflammatory leukotriene production (Mayser et al., 2002; Paine et al., 2023). Additionally, THH-FMT-mediated restoration of glutathione, an important antioxidant, alleviates oxidative stress, which synergizes with the normalization of AA metabolism to reduce lipid peroxidation and inflammatory cytokine secretion (Sorokin et al., 2018). This microbiota-AA metabolism crosstalk highlights the potential of gut-targeted interventions to modulate systemic inflammatory cascades in psoriasis.

### 4.3 Primary bile acid biosynthesis

As the end products of hepatic cholesterol metabolism, bile acids play essential roles in the emulsification and absorption of dietary lipids, as well as in the regulation of glucose and lipid metabolism. They can modulate the cytokine expression of skin immune cells like keratinocytes, dendritic cells, and macrophages by activating the farnesoid X receptor (FXR) and the G protein-coupled bile acid receptor 1 (GPBAR1/TGR5) (Cai et al., 2022; Liu and Wang, 2019; Portincasa et al., 2020; Yu et al., 2023; Ticho et al., 2019). During enterohepatic circulation, primary BAs synthesized in the liver are metabolized by intestinal microbial flora into secondary BAs, which exhibit anti-inflammatory properties and maintain intestinal barrier integrity. (di Gregorio et al., 2021; Foley et al., 2019; Hofmann and Hagey, 2008; Shi et al., 2022). In the pathological state of psoriasis, patients exhibit dysregulation of conjugated primary bile acids and glycodeoxycholic acid (Sorokin et al., 2018; Pietrzak et al., 2020), accompanied by an abnormal elevation of plasma cholesterol precursors. Microbiota dysbiosis reduces secondary BA production like UDCA/CDCA, leading to impaired FXR/TGR5 signaling and increased IL-17A production. Experiments have confirmed that the oral administration of secondary bile acids can reduce IL-17A production in IL-23-stimulated mouse T cells, inhibit CCL20 expression in keratinocytes, and decrease CCR6-positive Jurkat cells *via* TGR5/FXR-independent pathways, significantly improving symptoms of psoriatic dermatitis without obvious hepatotoxicity (Shi et al., 2022). Additionally, SCFAs, metabolites of gut microbiota, indirectly regulate skin inflammation by inhibiting IL-17 production in γδT cells (Dupraz et al., 2021) and activating the FXR/NF-κB pathway (Chen et al., 2023). *In vitro* supplementation with Bifidobacterium breve CCFM683 can restore the intestinal microbial flora of psoriasis model rats, promote cholic acid production, regulate the FXR/NF-κB pathway, reduce pro-inflammatory cytokine secretion, modulate keratinocytes, and maintain epidermal barrier function to alleviate PSO(75).

Our present study revealed that THH and THH-FMT regulate primary BA biosynthesis by targeting three key genera, One is, *Alistipes*, whose overgrowth in psoriasis mice disrupts glycine-conjugated BA production and enterohepatic circulation, and the THH-mediated reduction of Alistipes restores BA conjugation and improves antioxidant capacity (*via* increased Carnosine levels). The other two are *Akkermansia* and Ruminococcaceae, whose depletion in psoriasis mice correlates with reduced cholesterol and S1P levels. Restoration of these genera by THH likely enhances cholesterol supply for BA synthesis and promotes secondary BA production, which acts on skin immune cells through two pathways: (TGR5 activation inhibits IL-17A production in T cells and CCL20 expression in keratinocytes, reducing CCR6-positive immune cell infiltration into lesion, and FXR activation suppresses NF-κB signaling, thereby reducing pro-inflammatory cytokine (IL-6, TNF-α) secretion. This gut microbiota-BA-FXR/TGR5 axis provides a novel mechanism by which THH ameliorates psoriasis through distal regulation of skin inflammation.

### 4.4 Steroid hormone biosynthesis

Cholesterol serves as the primary precursor for steroid hormones, which exert anti-inflammatory and immunomodulatory effects in psoriasis (Sarkar et al., 2017; Binayke et al., 2025). Psoriatic lesions display localized cortisol deficiency, which impairs negative feedback on immune responses (Sarkar et al., 2017). Furthermore, reduced levels of testosterone and estrogen correlate with disease severity (Binayke et al., 2025; Kanda and Watanabe, 2005). Additionally, intestinal microbial flora regulate steroid hormone biosynthesis by modulating intestinal lipid metabolism and cholesterol availability. Our present results demonstrate that THH and THH-FMT restore steroid hormone balance by targeting Acetatifactor and *Akkermansia*. Depletion of these genera in psoriasis mice leads to impaired intestinal lipid metabolism, reduced cholesterol synthesis, and insufficient steroid hormone precursors. THH-mediated restoration of Acetatifactor and *Akkermansia* enhances cholesterol supply through two mechanisms. Modulating lipid metabolic pathways to increase cholesterol biosynthesis; and repairing the intestinal barrier *via Akkermansia* to reduce cholesterol loss. The subsequent normalization of steroid hormone levels like progesterone and aldosterone, directly suppresses Th17 differentiation and IL-17A/IL-6 secretion (Binayke et al., 2025), alleviating cutaneous inflammation. This finding highlights the role of intestinal microbial flora in regulating steroid hormone metabolism and provides a potential target for psoriasis therapy.

### 4.5 vitamin B6 metabolism and drug metabolism

Vitamin B6 (particularly its active form PLP) plays a protective role in psoriasis by inhibiting neutrophil infiltration, reducing oxidative stress, and suppressing NF-κB activity (Bai et al., 2025; Ueland et al., 2015). Psoriasis patients exhibit reduced serum PLP levels and an increased 4-PA/PLP ratio (Bai et al., 2025),which correlates with disease severity. Vitamin B6 is obtained from the diet and gut microbiota (Said, 2015), indicating a potential link between microbial dysbiosis and vitamin B6 deficiency in psoriasis. Our study identified a positive correlation between Pyridoxamine (a Vitamin B6 vitamer) and beneficial genera, such as *Akkermansia* and Alloprevotella, as well as a negative correlation with the PASI score and pro-inflammatory cytokines (IL-17A, IL-6, IL-1β). The THH and THH-FMT interventions restored both Pyridoxamine levels and the abundance of these beneficial genera, suggesting that THH modulates the gut microbiota-Vitamin B6 axis to suppress systemic immune overactivation. Mechanistically, Pyridoxamine, as a precursor of PLP, enhances antioxidant capacity and inhibits NF-κB signaling, thereby reducing oxidative stress-induced skin damage and inflammatory cytokine secretion. These findings support the notion that gut microbiota-targeted interventions can restore vitamin metabolism to alleviate psoriasis.

### 4.6 Drug metabolism: gut microbiota-mediated biotransformation of THH components *via* CYP450 and glutathione pathways

A critical finding of our integrated microbiome-metabolome analysis is the coordinated enrichment of the microbial secondary metabolite biosynthesis and drug metabolism-cytochrome P450 (CYP450) pathways, which highlights the importance of intestinal microbial flora in the biotransformation of THH components. THH contains over 120 natural compounds like triptolide, celastrol and wilforgine (Zhao et al., 2021), whose bioactivity and toxicity are highly dependent on metabolic modifications by intestinal microbial flora and host enzymes. We propose a synergistic mechanism for THH metabolism involving the microbiota-CYP450-glutathione pathway. Intestinal microbial flora biotransformation: Commensal bacteria convert THH components through hydrolysis, reduction, and dehydroxylation reactions. For example, triptolide undergoes de-epoxidation by Intestinal microbial flora to enhance its anti-inflammatory activity, while celastrol is reduced to alleviate oxidative damage (Zhao et al., 2021). This biotransformation step is critical for improving the bioavailability of THH components, as many natural compounds are poorly soluble or inactive in their parent form. THH components are further metabolized by CYP450 enzymes during enterohepatic circulation. CYP3A4 and CYP2C19 catalyze the epoxidation and ring-opening of Triptolide, producing derivatives with enhanced anti-inflammatory effects and reduced hepatotoxicity. Celastrol is metabolized by CYP1A2 and CYP2E1 to alleviate oxidative stress, while THH alkaloids undergo N-dealkylation *via* CYP2D6 to exert immunomodulatory effects. The metabolism of Vitamin B6, which is enriched in our study, provides PLP as a cofactor for CYP450 enzymes, thereby enhancing their catalytic activity. Additionally, THH-FMT-enriched glutathione metabolism scavenges reactive oxygen species (ROS) induced by Triptolide, synergizing with CYP450 to diminish hepatotoxicity. This integrated metabolic network not only elucidates the therapeutic efficacy of THH but also underscores the role of intestinal microbial flora in optimizing the ‘efficacy-toxicity ratio’ of natural products. By mediating the biotransformation of THH components, intestinal microbial flora enhances their anti-inflammatory activity while mitigating adverse effects, thereby providing a novel rationale for the clinical application of THH in psoriasis.

In conclusion, this study systematically investigates the specific interaction mechanisms of intestinal microbial flora in psoriasis and the intrinsic regulatory mechanisms of THH on the intestinal microbial flora of psoriatic mice through 16S rRNA gene sequencing analysis combined with intestinal content metabolomics. The 16S rRNA gene sequencing analysis reveals that psoriasis is associated with 13 distinct intestinal microbial flora at the genus level, which are primarily involved in the circulatory system, xenobiotics biodegradation and metabolism, metabolism of other amino acids, lipid metabolism, and carbohydrate metabolism. The intestinal contents metabolomics analysis identifies associations between IMQ-induced psoriatic mice and 445 differential metabolites, along with four potential metabolic pathways. These metabolites and pathways are primarily involved in arachidonic acid metabolism, taurine and hypotaurine metabolism, sphingolipid metabolism, and primary bile acid biosynthesis. However, THH mediates these microbial alterations to regulate metabolite levels, thereby achieving therapeutic effects against psoriasis. Our FMT study in THH-treated mice further demonstrates that THH can mediate 13 specific microorganisms and their associated metabolites involved in arachidonic acid metabolism, sphingolipid metabolism, and primary bile acid biosynthesis. Additionally, both THH and THHFMT can reduce intrinsic toxicity and exert therapeutic effects through steroid hormone biosynthesis, vitamin B6 metabolism, and drug metabolism.

Several limitations of this study warrant acknowledgment. Our FMT experiment lacked a vehicle-FMT control (healthy donor microbiota); thus, the therapeutic effect of THHFMT cannot be unequivocally attributed to THH-reshaped microbiota rather than a general reconstitution effect. In the co-housing experiment, microbial cross-colonization was not verified *via* 16S sequencing; as a result, the observed phenotypic improvement might be confounded by other cage-sharing effects (e.g., stress, coprophagy). Although co-housing has been shown to transfer disease-associated microbiota and phenotypes, for instance, inflammatory bowel disease (IBD) microbiota can transfer pathogenic traits to wild-type mice (Leber et al., 2018). Mice with IBD can transfer pathogenic microbiota and colitis susceptibility to wild-type healthy mice. The microbiota from inflammatory bowel disease mice lacking estrogen receptor β (ERβ) (Ma et al., 2022) can exacerbate colitis and anxiety-like behaviors in wild-type mice through co-housing. Healthy mice co-housed with Alzheimer’s disease (AD) model mice acquire similar dysbacteriosis, leading to increased levels of brain amyloid-β42 and cognitive impairment (Yang et al., 2026; Li et al., 2024). However, direct evidence of such transfer within our specific model remains absent. Additionally, functional pathways were predicted from 16S data using PICRUSt2, a method that is inherently computational and constrained by database coverage and strain-level resolution; consequently, our interpretations of these pathways should be regarded as hypothesis-generating. Future investigations incorporating healthy-donor FMT controls, direct verification of microbial colonization, and shotgun metagenomic analysis are warranted.

## Data Availability

The raw data have been deposited in the NCBI Sequence Read Archive (SRA) under BioProject accession number PRJNA1460424. The original contributions presented in the study are included in the article/Supplementary Material, further inquiries can be directed to the corresponding authors.

## References

[B1] Bai R. Cheng X. Yang Y. Zhang J. Tian Q. (2025). Vitamin B6 catabolism and psoriasis risk: a cross-sectional study. Clin. Exp. Dermatol 50, 1366–1372. 10.1093/ced/llaf065 39938060

[B2] Binayke A. Dalal R. Suri C. Dandotiya J. Sadhu S. Kumar Y. (2025). Testosterone suppresses IL-17 expression by targeting RORgammat functions. Eur. J. Immunol. 55, e70016. 10.1002/eji.70016 40760856

[B3] Bochenska K. Gabig-Ciminska M. (2020). Unbalanced sphingolipid metabolism and its implications for the pathogenesis of psoriasis. Molecules 25, 25. 10.3390/molecules25051130 32138315 PMC7179243

[B4] Cai J. Rimal B. Jiang C. Chiang J. Y. L. Patterson A. D. (2022). Bile acid metabolism and signaling, the microbiota, and metabolic disease. Pharmacol. Ther. 237, 108238. 10.1016/j.pharmthera.2022.108238 35792223

[B5] Cai J. Zhou X. Zhuang Y. Cui L. Ma R. Chen Y. (2025). Reprogramming of fatty acid metabolism *via* PPARalpha-orchestrated FADS2 in keratinocytes modulates skin inflammation in psoriasis. Adv. Sci. (Weinh) 12, e17049. 10.1002/advs.202417049 40878384 PMC12561275

[B6] Chen X. Chen Y. Stanton C. Ross R. P. Zhao J. Chen W. (2023). Dose-response efficacy and mechanisms of orally administered Bifidobacterium breve CCFM683 on IMQ-induced psoriasis in mice. Nutrients 15, 15. 10.3390/nu15081952 37111171 PMC10143451

[B7] Cho Y. Lew B. L. Seong K. Kim N. I. (2004). An inverse relationship between ceramide synthesis and clinical severity in patients with psoriasis. J. Korean Med. Sci. 19, 859–863. 10.3346/jkms.2004.19.6.859 15608398 PMC2816304

[B8] di Gregorio M. C. Cautela J. Galantini L. (2021). Physiology and physical chemistry of bile acids. Int. J. Mol. Sci. 22, 1780. 10.3390/ijms22041780 33579036 PMC7916809

[B9] Douglas G. M. Maffei V. J. Zaneveld J. R. Yurgel S. N. Brown J. R. Taylor C. M. (2020). PICRUSt2 for prediction of metagenome functions. Nat. Biotechnol. 38, 685–688. 10.1038/s41587-020-0548-6 32483366 PMC7365738

[B10] Dupraz L. Magniez A. Rolhion N. Richard M. L. Da Costa G. Touch S. (2021). Gut microbiota-derived short-chain fatty acids regulate IL-17 production by mouse and human intestinal gammadelta T cells. Cell Rep. 36, 109332. 10.1016/j.celrep.2021.109332 34233192

[B11] Ely P. H. (2018). Is psoriasis a bowel disease? Successful treatment with bile acids and bioflavonoids suggests it is. Clin. Dermatol 36, 376–389. 10.1016/j.clindermatol.2018.03.011 29908580

[B12] Foley M. H. O'Flaherty S. Barrangou R. Theriot C. M. (2019). Bile salt hydrolases: gatekeepers of bile acid metabolism and host-microbiome crosstalk in the gastrointestinal tract. PLoS Pathog. 15, e1007581. 10.1371/journal.ppat.1007581 30845232 PMC6405046

[B13] Forsgren M. Isolauri E. Salminen S. Rautava S. (2017). Late preterm birth has direct and indirect effects on infant gut microbiota development during the first six months of life. Acta Paediatr. 106, 1103–1109. 10.1111/apa.13837 28316118 PMC5763336

[B14] Fouad N. Mostafa F. Soltan M. Zaki A. Hassan R. A. (2022). Skin colonization of *Staphylococcus aureus* harboring superantigen toxin genes and its correlation with serum IL-22 level in psoriasis patients. Egypt J. Immunol. 29, 94–105. 36198107

[B15] Fu J. Li G. Li X. Song S. Cheng L. Rui B. (2024). Gut commensal *Alistipes* as a potential pathogenic factor in colorectal cancer. Discov. Oncol. 15, 473. 10.1007/s12672-024-01393-3 39331213 PMC11436608

[B16] Gao Q. Tian W. Yang H. Hu H. Zheng J. Yao X. (2024). Shen-Ling-Bai-Zhu-San alleviates the imbalance of intestinal homeostasis in dextran sodium sulfate-induced colitis mice by regulating gut microbiota and inhibiting the NLRP3 inflammasome activation. J. Ethnopharmacol. 319, 117136. 10.1016/j.jep.2023.117136 37704122

[B17] Greb J. E. Goldminz A. M. Elder J. T. Lebwohl M. G. Gladman D. D. Wu J. J. (2016). Psoriasis 2016. Nat. Rev. Dis. Prim. 2, 16082. 10.1038/nrdp.2016.82 27883001

[B18] Hofmann A. F. Hagey L. R. (2008). Bile acids: chemistry, pathochemistry, biology, pathobiology, and therapeutics. Cell Mol. Life Sci. 65, 2461–2483. 10.1007/s00018-008-7568-6 18488143 PMC11131813

[B19] Hong D. Xiong H. Lu S. Ma J. Shi Z. (2025). Metabolic regulation of the immune cell in psoriasis: mechanisms and interventions. Curr. Opin. Immunol. 96, 102614. 10.1016/j.coi.2025.102614 40674835

[B20] Kahleova H. Rembert E. Alwarith J. Yonas W. N. Tura A. Holubkov R. (2020). Effects of a low-fat vegan diet on gut microbiota in overweight individuals and relationships with body weight, body composition, and insulin sensitivity. A randomized clinical trial. Nutrients 12, 12. 10.3390/nu12102917 32987642 PMC7598634

[B21] Kanda N. Watanabe S. (2005). Regulatory roles of sex hormones in cutaneous biology and immunology. J. Dermatol Sci. 38, 1–7. 10.1016/j.jdermsci.2004.10.011 15795118

[B22] Kelly D. Conway S. Aminov R. (2005). Commensal gut bacteria: mechanisms of immune modulation. Trends Immunol. 26, 326–333. 10.1016/j.it.2005.04.008 15922949

[B23] Kim J. H. Lee S. R. Ahn H. K. Hong H. T. Jo U. H. Im J. P. (2025). A chronic psoriasis model using long-term imiquimod application in IL-10-deficient mice: recapitulating skin inflammation, comorbidities, and gut-skin axis alterations. Ann. Dermatol 37, 383–396. 10.5021/ad.25.108 41331719 PMC12715878

[B24] Korman N. J. (2020). Management of psoriasis as a systemic disease: what is the evidence? Br. J. Dermatol 182, 840–848. 10.1111/bjd.18245 31225638 PMC7187293

[B25] Kozlowska D. Harasim-Symbor E. Mysliwiec H. Milewska A. J. Chabowski A. Flisiak I. (2019). Serum sphingolipid level in psoriatic patients with obesity. Postepy Dermatol Alergol. 36, 714–721. 10.5114/ada.2019.91422 31998000 PMC6986291

[B26] Kuang Y. S. Li S. H. Guo Y. Lu J. H. He J. R. Luo B. J. (2016). Composition of gut microbiota in infants in China and global comparison. Sci. Rep. 6, 36666. 10.1038/srep36666 27827448 PMC5101483

[B27] Leber A. Hontecillas R. Tubau-Juni N. Zoccoli-Rodriguez V. Abedi V. Bassaganya-Riera J. (2018). NLRX1 modulates immunometabolic mechanisms controlling the host-gut microbiota interactions during inflammatory bowel disease. Front. Immunol. 9, 363. 10.3389/fimmu.2018.00363 29535731 PMC5834749

[B28] Lebwohl M. (2018). Psoriasis. Ann. Intern Med. 168, ITC49–ITC64. 10.7326/AITC201804030 29610923

[B29] Lee M. T. Tan X. Le H. H. Besler K. Thompson S. Harris-Tryon T. (2024). Gut bacterial sphingolipid production modulates dysregulated skin lipid homeostasis. bioRxiv, 2024.12.29.629238. 10.1101/2024.12.29.629238 39803564 PMC11722302

[B30] Li Q. Fang H. Dang E. Wang G. (2020). The role of ceramides in skin homeostasis and inflammatory skin diseases. J. Dermatol Sci. 97, 2–8. 10.1016/j.jdermsci.2019.12.002 31866207

[B31] Li N. Liang S. Chen Q. Zhao L. Li B. Huo G. (2021). Distinct gut microbiota and metabolite profiles induced by delivery mode in healthy Chinese infants. J. Proteomics 232, 104071. 10.1016/j.jprot.2020.104071 33307251

[B32] Li J. Shan W. Zuo Z. (2024). Co-housing with Alzheimer's disease mice induces changes in gut microbiota and impairment of learning and memory in control mice. CNS Neurosci. Ther. 30, e14491. 10.1111/cns.14491 37789692 PMC11017403

[B33] Li J. Xu J. Guo X. Xu H. Huang C. Nie Y. (2025). Odoribacter splanchnicus-A next-generation probiotic candidate. Microorganisms 13, 13. 10.3390/microorganisms13040815 40284651 PMC12029356

[B34] Lin Z. Ye W. Zu X. Xie H. Li H. Li Y. (2018). Integrative metabolic and microbial profiling on patients with spleen-yang-deficiency syndrome. Sci. Rep. 8, 6619. 10.1038/s41598-018-24130-7 29700349 PMC5920061

[B35] Liou J. M. Chen C. C. Chang C. M. Fang Y. J. Bair M. J. Chen P. Y. (2019). Long-term changes of gut microbiota, antibiotic resistance, and metabolic parameters after *Helicobacter pylori* eradication: a multicentre, open-label, randomised trial. Lancet Infect. Dis. 19, 1109–1120. 10.1016/S1473-3099(19)30272-5 31559966

[B36] Liu Q. (2011). Triptolide and its expanding multiple pharmacological functions. Int. Immunopharmacol. 11, 377–383. 10.1016/j.intimp.2011.01.012 21255694

[B37] Liu X. Wang Y. (2019). An overview of bile acid synthesis and its physiological and pathological functions. Yi Chuan 41, 365–374. 10.16288/j.yczz.19-011 31106772

[B38] Lu S. Wang Q. Li G. Sun S. Guo Y. Kuang H. (2015). The treatment of rheumatoid arthritis using Chinese medicinal plants: from pharmacology to potential molecular mechanisms. J. Ethnopharmacol. 176, 177–206. 10.1016/j.jep.2015.10.010 26471289

[B39] Lu W. Deng Y. Fang Z. Zhai Q. Cui S. Zhao J. (2021). Potential role of probiotics in ameliorating psoriasis by modulating gut microbiota in imiquimod-induced psoriasis-like mice. Nutrients 13, 13. 10.3390/nu13062010 34207960 PMC8230682

[B40] Mysliwiec H. Baran A. Harasim-Symbor E. Choromanska B. Mysliwiec P. Milewska A. J. (2017). Increase in circulating sphingosine-1-phosphate and decrease in ceramide levels in psoriatic patients. Arch. Dermatol Res. 309, 79–86. 10.1007/s00403-016-1709-9 27988894 PMC5309277

[B41] Ma Y. Liu T. Li X. Kong A. Xiao R. Xie R. (2022). Estrogen receptor β deficiency impairs gut microbiota: a possible mechanism of IBD-induced anxiety-like behavior. Microbiome 10, 160. 10.1186/s40168-022-01356-2 36175956 PMC9520828

[B42] Mao M. Yuan Y. Li R. Kuang Y. Lu Y. Zhu W. (2025). Modulation of gut propionate and intestinal mucosal protection by *Bifidobacterium longum*: mitigating methotrexate side effects without compromising the efficacy of psoriasis therapy. Int. Immunopharmacol. 149, 114196. 10.1016/j.intimp.2025.114196 39904035

[B43] Masuda-Kuroki K. Alimohammadi S. Di Nardo A. (2023). The role of sphingolipids and sphingosine-1-phosphate-sphingosine-1-phosphate-receptor signaling in psoriasis. Cells 12, 12. 10.3390/cells12192352 37830566 PMC10571972

[B44] Mayser P. Grimm H. Grimminger F. (2002). n-3 fatty acids in psoriasis. Br. J. Nutr. 87 (Suppl. 1), S77–S82. 10.1079/bjn2001459 11895157

[B45] Mobeen F. Sharma V. Tulika P. (2018). Enterotype variations of the healthy human gut microbiome in different geographical regions. Bioinformation 14, 560–573. 10.6026/97320630014560 31223215 PMC6563668

[B46] Morin S. Tremblay A. Dumais E. Julien P. Flamand N. Pouliot R. (2023). Eicosapentaenoic acid influences the lipid profile of an *in vitro* psoriatic skin model produced with T cells. Biomolecules 13, 13. 10.3390/biom13091413 37759812 PMC10526348

[B47] Nakajima K. Terao M. Takaishi M. Kataoka S. Goto-Inoue N. Setou M. (2013). Barrier abnormality due to ceramide deficiency leads to psoriasiform inflammation in a mouse model. J. Invest Dermatol 133, 2555–2565. 10.1038/jid.2013.199 23633022

[B48] Paine A. Brookes P. S. Bhattacharya S. Li D. De La Luz Garcia-Hernandez M. Tausk F. (2023). Dysregulation of bile acids, lipids, and nucleotides in psoriatic arthritis revealed by unbiased profiling of serum metabolites. Arthritis Rheumatol. 75, 53–63. 10.1002/art.42288 35818333 PMC9797425

[B49] Pietrzak A. Chabros P. Grywalska E. Pietrzak D. Kandzierski G. Wawrzycki B. O. (2020). Serum concentration of interleukin 6 is related to inflammation and dyslipidemia in patients with psoriasis. Postepy Dermatol Alergol. 37, 41–45. 10.5114/ada.2018.78028 32467682 PMC7247055

[B50] Portincasa P. Di Ciaula A. Garruti G. Vacca M. De Angelis M. Wang D. Q. (2020). Bile acids and GPBAR-1: dynamic interaction involving genes, environment and gut microbiome. Nutrients 12, 12. 10.3390/nu12123709 33266235 PMC7760347

[B51] Puig L. Costanzo A. Munoz-Elias E. J. Jazra M. Wegner S. Paul C. F. (2022). The biological basis of disease recurrence in psoriasis: a historical perspective and current models. Br. J. Dermatol 186, 773–781. 10.1111/bjd.20963 34939663 PMC9374062

[B52] Ramirez-Bosca A. Navarro-Lopez V. Martinez-Andres A. Such J. Frances R. Horga de la Parte J. (2015). Identification of bacterial DNA in the peripheral blood of patients with active psoriasis. JAMA Dermatol 151, 670–671. 10.1001/jamadermatol.2014.5585 25760018

[B53] Said H. M. (2015). Water-soluble vitamins. World Rev. Nutr. Diet. 111, 30–37. 10.1159/000362294 25418386

[B54] Sarkar M. K. Kaplan N. Tsoi L. C. Xing X. Liang Y. Swindell W. R. (2017). Endogenous glucocorticoid deficiency in psoriasis promotes inflammation and abnormal differentiation. J. Invest Dermatol 137, 1474–1483. 10.1016/j.jid.2017.02.972 28259685 PMC5545780

[B55] Scher J. U. Ubeda C. Artacho A. Attur M. Isaac S. Reddy S. M. (2015). Decreased bacterial diversity characterizes the altered gut microbiota in patients with psoriatic arthritis, resembling dysbiosis in inflammatory bowel disease. Arthritis Rheumatol. 67, 128–139. 10.1002/art.38892 25319745 PMC4280348

[B56] Shao-Yu Y. Niu D. Chen J. Li W. Y. Wang X. Meng Q. W. (2025). Antibiotic cocktail-induced changes in gut microbiota drive alteration of bile acid metabolism to restrain Th17 differentiation through the FXR-NLRP3 axis. Gut Microbes 17, 2582944. 10.1080/19490976.2025.2582944 41305918 PMC12667630

[B57] Shi Z. Wu X. Wu C. Y. Singh S. P. Law T. Yamada D. (2022). Bile acids improve psoriasiform dermatitis through inhibition of IL-17A expression and CCL20-CCR6-mediated trafficking of T cells. J. Invest Dermatol 142, 1381–90 e11. 10.1016/j.jid.2021.10.027 34808237 PMC9728300

[B58] Simancas-Racines D. Roman-Galeano N. M. Verde L. Annunziata G. Marchetti M. Matos A. (2025). Targeting cytokine dysregulation in psoriasis: the role of dietary interventions in modulating the immune response. Int. J. Mol. Sci. 26, 26. 10.3390/ijms26072895 40243475 PMC11988797

[B59] Sorokin A. V. Domenichiello A. F. Dey A. K. Yuan Z. X. Goyal A. Rose S. M. (2018). Bioactive lipid mediator profiles in human psoriasis skin and blood. J. Invest Dermatol 138, 1518–1528. 10.1016/j.jid.2018.02.003 29454560 PMC6121727

[B60] Stecher B. (2015). The roles of inflammation, nutrient availability and the commensal microbiota in enteric pathogen infection. Microbiol. Spectr. 3, 3. 10.1128/microbiolspec.MBP-0008-2014 26185088

[B61] Sulthana S. Chary P. S. Bhavana V. Pardhi E. Singh S. B. Mehra N. K. (2023). Development and evaluation emulgel for effective management of the imiquimod-induced psoriasis. Inflammopharmacology 31, 301–320. 10.1007/s10787-022-01131-7 36609718

[B62] Sun Y. Wang J. Hu P. Tang Y. Wang Y. Ye J. (2024). Molecular mechanism through which *Tripterygium hypoglaucum* (Levl.) Hutch alleviates psoriasis. Biomed. Pharmacother. 181, 117647. 10.1016/j.biopha.2024.117647 39504627

[B63] Takeshita J. Grewal S. Langan S. M. Mehta N. N. Ogdie A. Van Voorhees A. S. (2017). Psoriasis and comorbid diseases: epidemiology. J. Am. Acad. Dermatol 76, 377–390. 10.1016/j.jaad.2016.07.064 28212759 PMC5731650

[B64] Thio H. B. (2018). The microbiome in psoriasis and psoriatic arthritis: the skin perspective. J. Rheumatol. Suppl. 94, 30–31. 10.3899/jrheum.180133 29858350

[B65] Thursby E. Juge N. (2017). Introduction to the human gut microbiota. Biochem. J. 474, 1823–1836. 10.1042/BCJ20160510 28512250 PMC5433529

[B66] Ticho A. L. Malhotra P. Dudeja P. K. Gill R. K. Alrefai W. A. (2019). Intestinal absorption of bile acids in health and disease. Compr. Physiol. 10, 21–56. 10.1002/cphy.c190007 31853951 PMC7171925

[B67] Tonon K. M. Morais T. B. Taddei C. R. Araujo-Filho H. B. Abrao A. Miranda A. (2021). Gut microbiota comparison of vaginally and cesarean born infants exclusively breastfed by mothers secreting alpha1-2 fucosylated oligosaccharides in breast milk. PLoS One 16, e0246839. 10.1371/journal.pone.0246839 33556125 PMC7870049

[B68] Ueland P. M. Ulvik A. Rios-Avila L. Midttun O. Gregory J. F. (2015). Direct and functional biomarkers of vitamin B6 status. Annu. Rev. Nutr. 35, 33–70. 10.1146/annurev-nutr-071714-034330 25974692 PMC5988249

[B69] Vahlquist C. Berne B. Boberg M. Michaelsson G. Vessby B. (1985). The fatty-acid spectrum in plasma and adipose tissue in patients with psoriasis. Arch. Dermatol Res. 278, 114–119. 10.1007/BF00409217 4096537

[B70] Wang J. Li W. Wang C. Wang L. He T. Hu H. (2020). Enterotype bacteroides is associated with a high risk in patients with diabetes: a pilot study. J. Diabetes Res. 2020, 6047145. 10.1155/2020/6047145 32064276 PMC6996672

[B71] Wang Q. Wang C. Abdullah T. W. Qiu Z. Song M. Cao Y. (2022a). Hydroxytyrosol alleviates dextran sulfate sodium-Induced colitis by modulating inflammatory responses, intestinal barrier, and microbiome. J. Agric. Food Chem. 70, 2241–2252. 10.1021/acs.jafc.1c07568 35133830

[B72] Wang X. Liu X. Xiao S. Zhang Z. Wu L. Cheng Y. (2022b). Comparison of gut microbiota compositions and corresponding genetic and metabolic features between guttate and plaque psoriasis by metagenomic sequencing. Microb. Pathog. 167, 105560. 10.1016/j.micpath.2022.105560 35504350

[B73] Wang Y. Zhao N. Meng Y. Chen J. Qi C. Hu X. (2024). Bcat2-Mediated branched-chain amino acid catabolism is linked to the aggravated inflammation in obese with psoriasis mice. Mol. Nutr. Food Res. 68, e2300720. 10.1002/mnfr.202300720 38581348

[B74] Wen C. Pan Y. Gao M. Wang J. Huang K. Tu P. (2023). Altered gut microbiome composition in nontreated plaque psoriasis patients. Microb. Pathog. 175, 105970. 10.1016/j.micpath.2023.105970 36621696

[B75] Wu F. Jiang X. Chen G. Zhang L. (2025). Integrated microbiome and metabolome analysis reveals microbial-metabolic interactions in psoriasis pathogenesis. BMC Microbiol. 25, 665. 10.1186/s12866-025-04395-5 41107739 PMC12532935

[B76] Xin J. He L. Li Y. Pu Q. Du X. Ban F. (2025). Sanguinarine chloride hydrate mitigates colitis symptoms in mice through the regulation of the intestinal microbiome and metabolism of short-chain fatty acids. Biochim. Biophys. Acta Mol. Basis Dis. 1871, 167579. 10.1016/j.bbadis.2024.167579 39561858

[B77] Xu J. Bjursell M. K. Himrod J. Deng S. Carmichael L. K. Chiang H. C. (2003). A genomic view of the human-bacteroides thetaiotaomicron symbiosis. Science 299, 2074–2076. 10.1126/science.1080029 12663928

[B78] Yang C. Qi W. Li W. Liang F. Wang H. Zhang Y. (2026). Gut microbiota transmission induces cognitive impairment through amyloid pathology in wild-type mice. Neuroscience 600, 104–111. 10.1016/j.neuroscience.2026.02.041 41759988

[B79] Yu H. Nie R. Shen C. (2023). The role of bile acids in regulating glucose and lipid metabolism. Endocr. J. 70, 359–374. 10.1507/endocrj.EJ22-0544 36928060

[B80] Yu Z. Wang Y. Guo Y. Zhu R. Fang Y. Yao Q. (2025). Exploring the therapeutic and gut microbiota-modulating effects of qingreliangxuefang on IMQ-induced psoriasis. Drug Des. Devel Ther. 19, 3269–3291. 10.2147/dddt.S492044 40322026 PMC12048299

[B81] Zhang X. Li N. Yao Y. Liang X. Qu X. Liu X. (2016). Identification of species in tripterygium (celastraceae) based on DNA barcoding. Biol. Pharm. Bull. 39, 1760–1766. 10.1248/bpb.b15-00956 27601081

[B82] Zhao J. Zhang F. Xiao X. Wu Z. Hu Q. Jiang Y. (2021). *Tripterygium hypoglaucum* (Levl.) hutch and its main bioactive components: recent advances in pharmacological activity, pharmacokinetics and potential toxicity. Front. Pharmacol. 12, 715359. 10.3389/fphar.2021.715359 34887747 PMC8650721

[B83] Zhao M. Chu J. Feng S. Guo C. Xue B. He K. (2023). Immunological mechanisms of inflammatory diseases caused by gut microbiota dysbiosis: a review. Biomed. Pharmacother. 164, 114985. 10.1016/j.biopha.2023.114985 37311282

[B84] Zheng J. Hu J. Yang Y. Xiong L. Yang H. Zhang Z. (2023). Suppressive effect of *Tripterygium hypoglaucum* (Levl.) Hutch extract on rheumatoid arthritis in mice by modulating inflammasome and bile acid metabolism. Biomed. Pharmacother. 167, 115494. 10.1016/j.biopha.2023.115494 37734264

[B85] Zhuang X. Li T. Li M. Huang S. Qiu Y. Feng R. (2019). Systematic review and meta-analysis: short-chain fatty acid characterization in patients with inflammatory bowel disease. Inflamm. Bowel Dis. 25, 1751–1763. 10.1093/ibd/izz188 31498864

