# Supplementary figures and images for: Therapeutic effect of Tripterygium hypoglaucum (H. Lév.) Hutch. extract on psoriasis-like skin inflammation correlated with gut microbiota homeostasis in mice
Source: Front Pharmacol. 2026 May 12;17:1822819. doi: 10.3389/fphar.2026.1822819 (PMC13201418; doi:10.3389/fphar.2026.1822819)

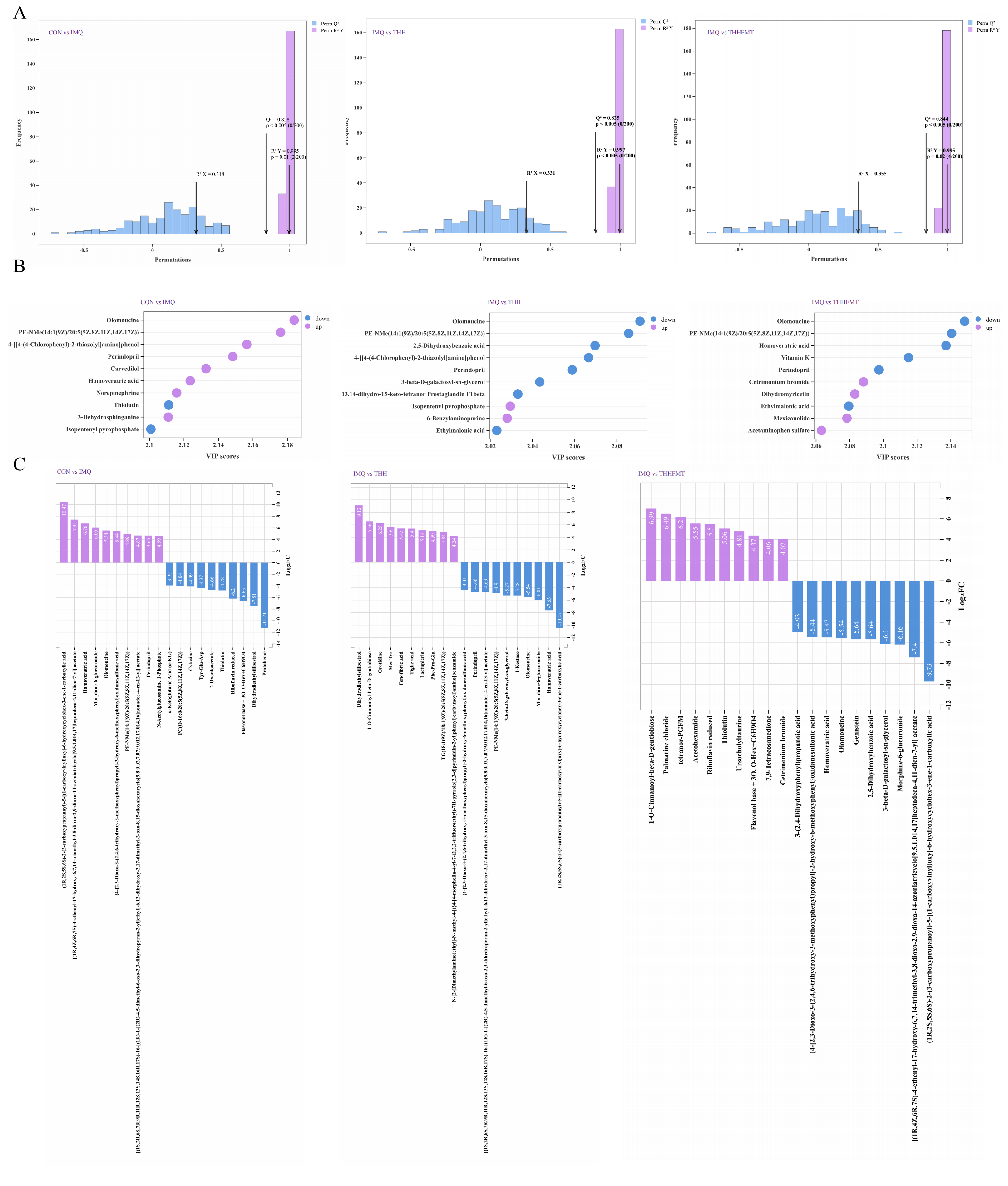

Supplement: Supplementary file 1 [file Image3.jpeg]

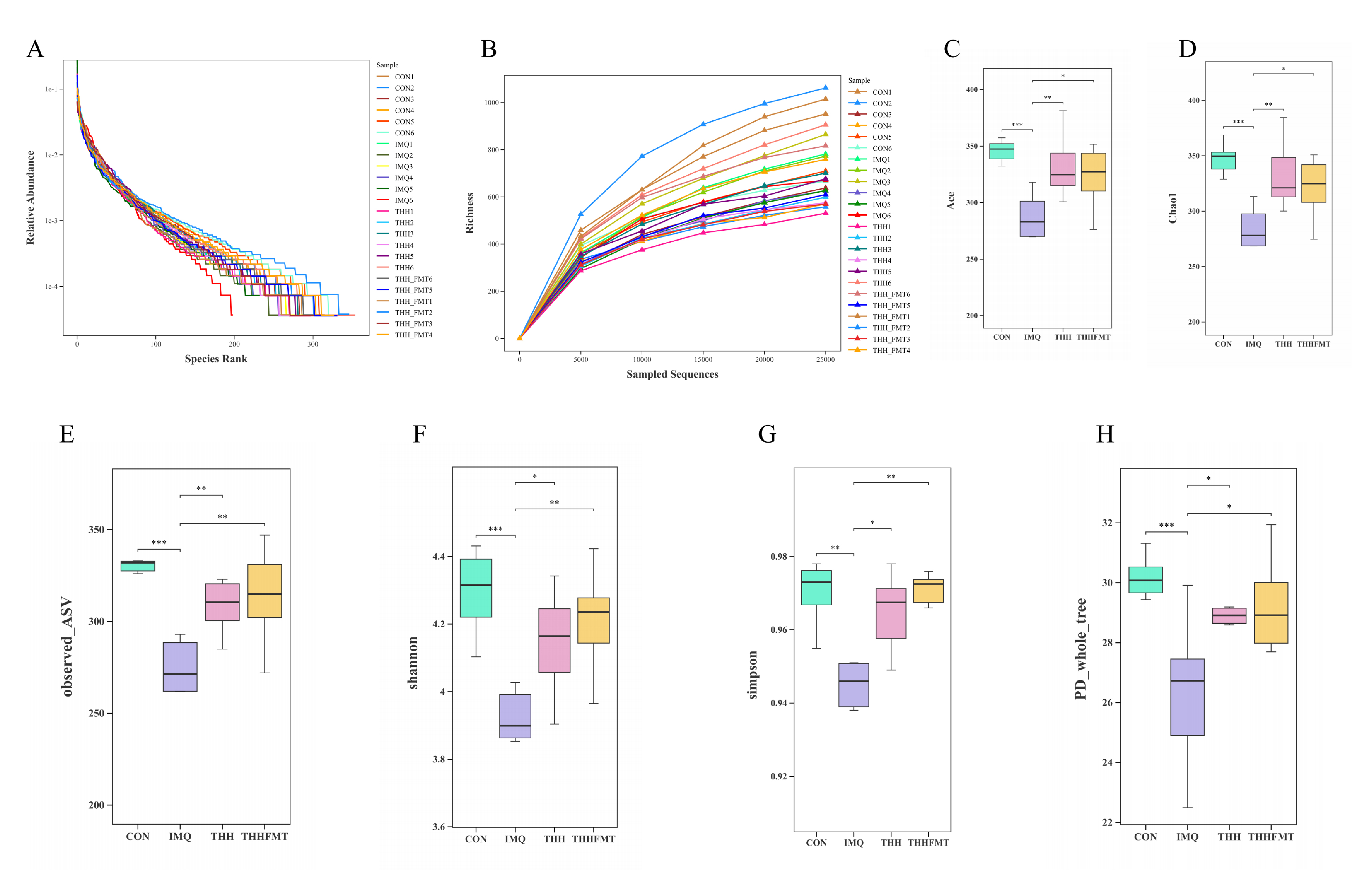

Supplement: Supplementary file 2 [file Image1.jpeg]

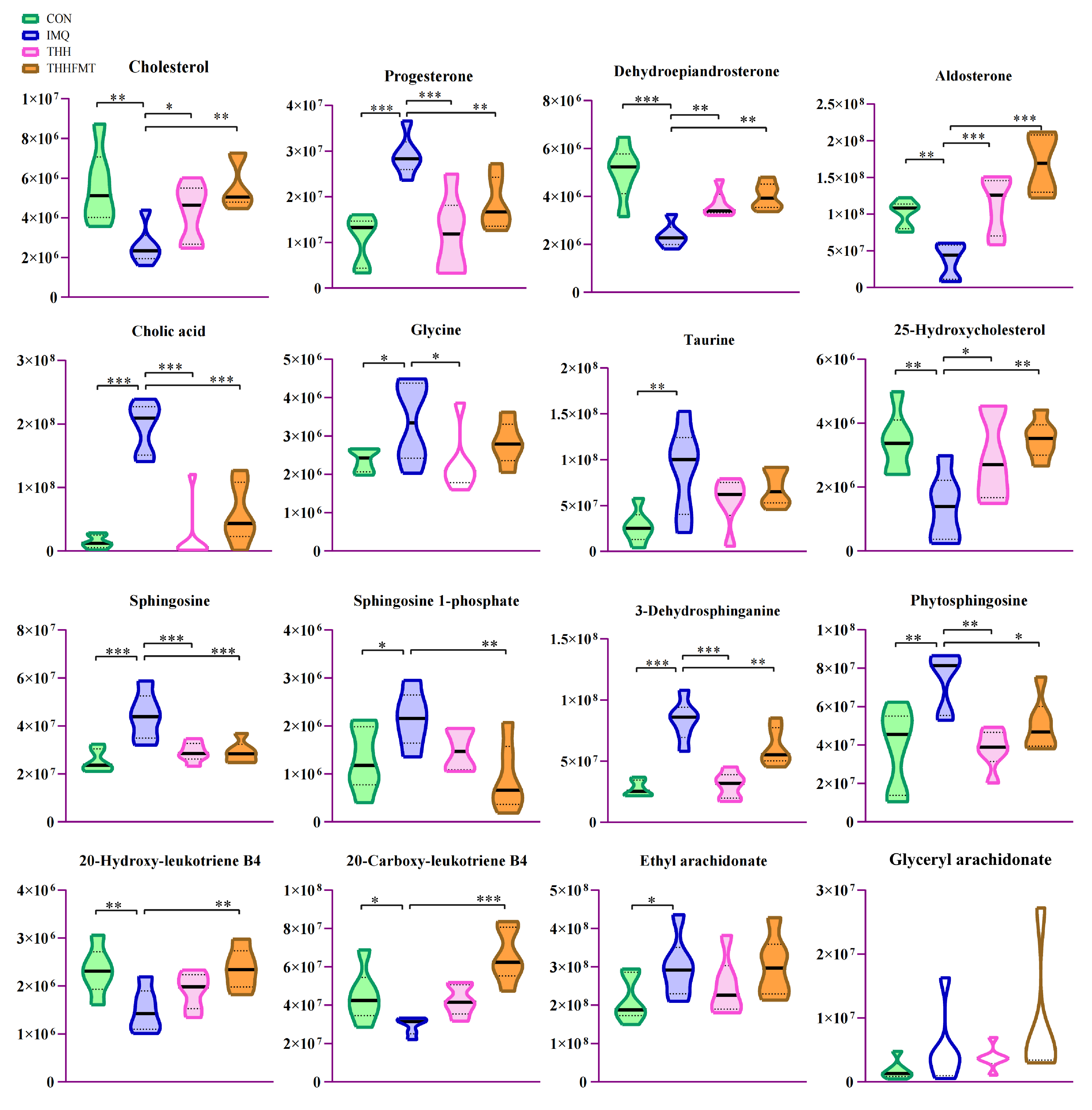

Supplement: Supplementary file 3 [file Image4.jpeg]

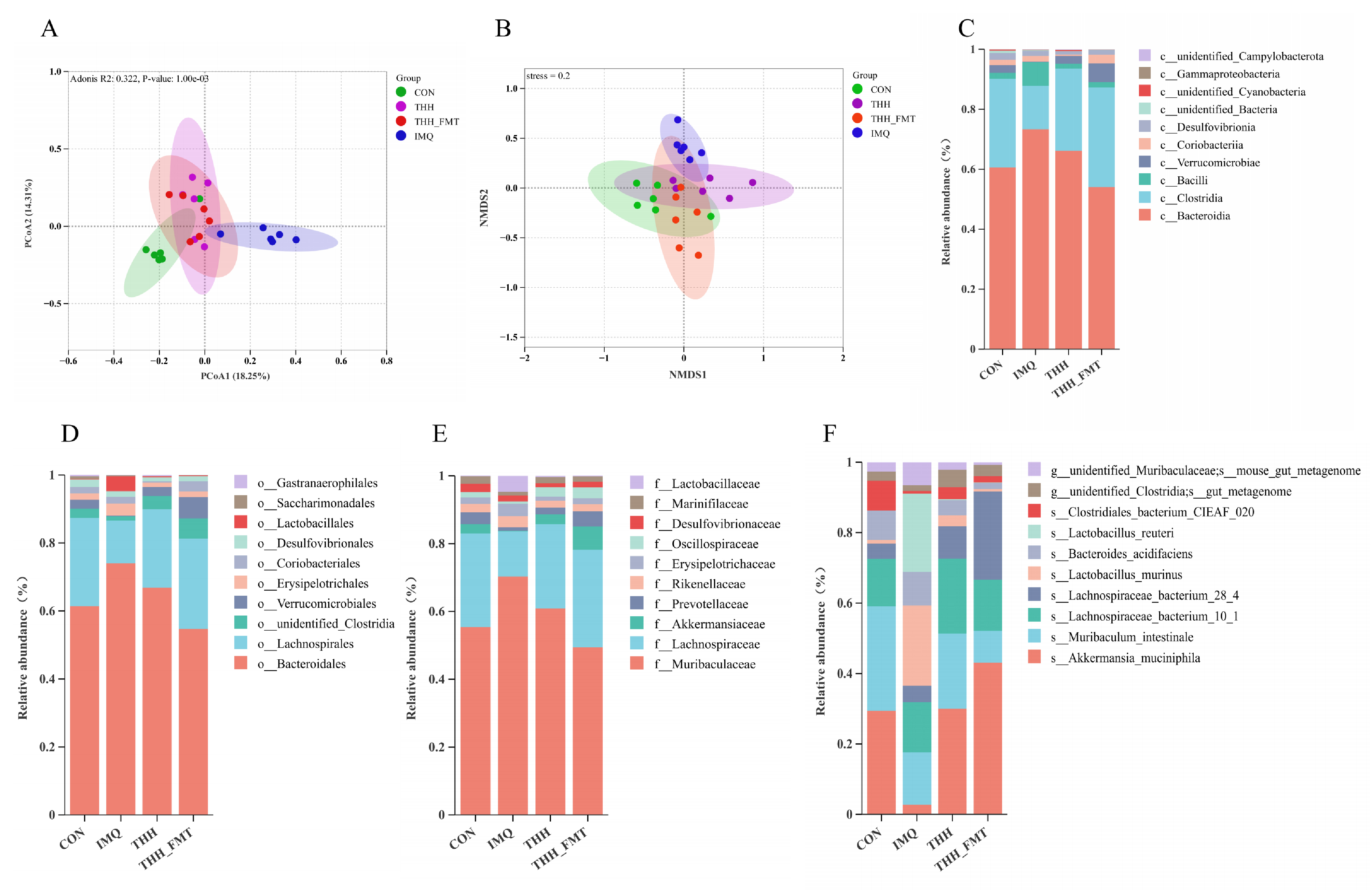

Supplement: Supplementary file 4 [file Image2.jpeg]

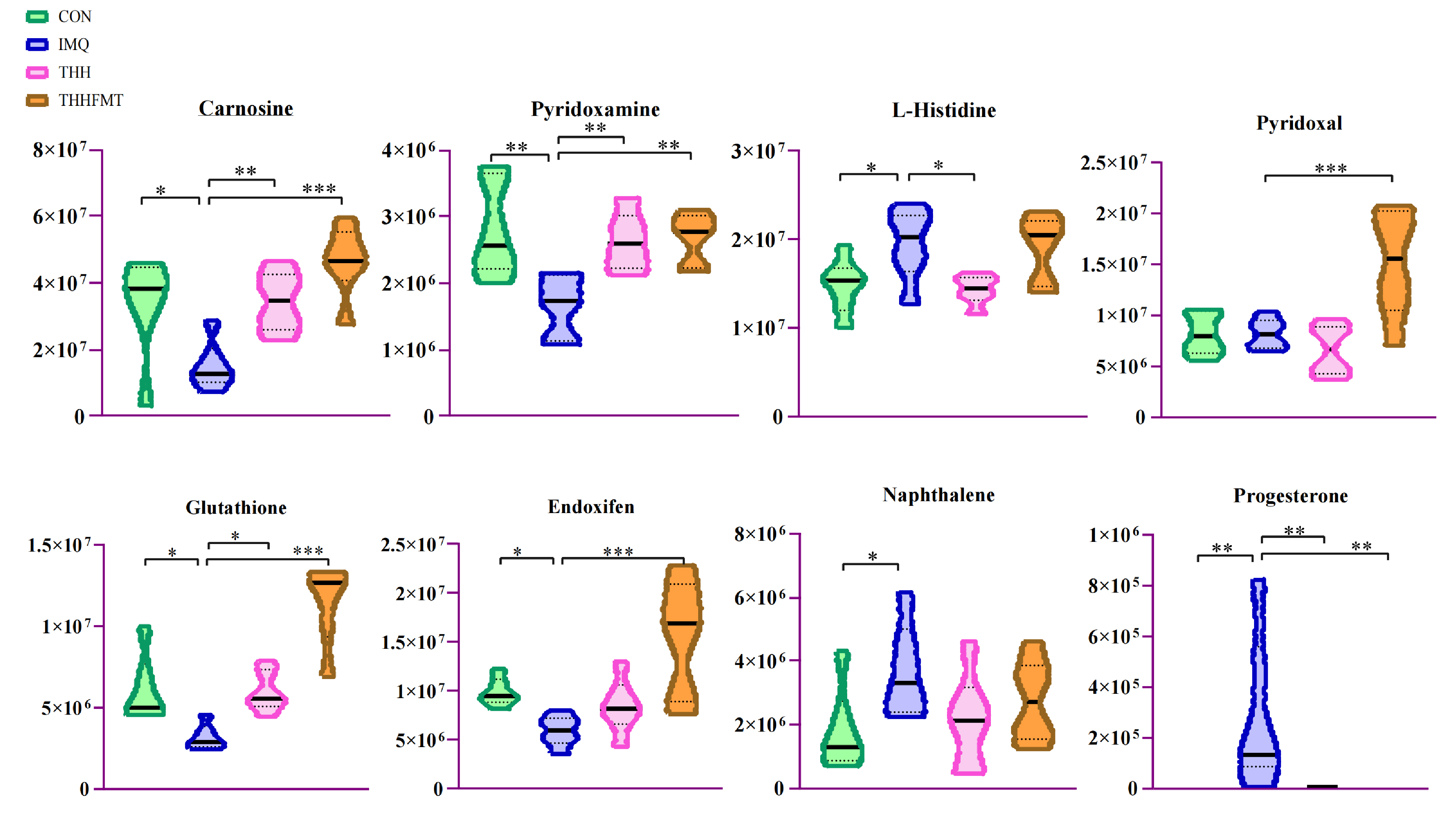

Supplement: Supplementary file 5 [file Image5.jpeg]
